# Rapid moving by liquid-amplified electrostatic rolling

**DOI:** 10.1126/sciadv.ady5143

**Published:** 2025-09-10

**Authors:** Fei Jia, Shengjun Fan, Jianglong Guo, Yanju Liu, Jinsong Leng

**Affiliations:** ^1^ Department of Astronautical Science and Mechanics, Harbin Institute of Technology, Harbin 150001, China.; ^2^ School of Science, Harbin Institute of Technology (Shenzhen), Shenzhen 518055, China.; ^3^ The Center for Composite Materials and Structures, Harbin Institute of Technology, Harbin 150080, China.

## Abstract

Mobile robots that simultaneously have fast speeds, sufficient load-carrying capabilities, and multiple locomotive functions have always been challenging to develop. Here, we introduce a liquid-amplified electrostatic rolling (LAER) mechanism, which elegantly integrates actuation and adhesion into a streamline single-degree-of-freedom structure. Based on this, we developed a rigid tethered LAER roller (0.015 grams) that exhibited a rapid moving speed of ~210 body length per second on ceilings and a flexible tethered roller (10.600 grams) that had a load-to-weight ratio of ~121 moving on the ground. Liquid regulating modules were embedded into LAER structures to exhibit controlled moving. We also developed a single-wheeled and two-wheeled untethered LAER robots with onboard power supply. Furthermore, we demonstrate a LAER linear actuator and a camera equipped untethered LAER robot for preliminary environmental monitoring. The lightweight, scalable LAER structure is promising to bring fast electrostatic actuators, motors, and robots with superior load-to-weight ratios and multimodal locomotion capabilities such as turning, circular moving, and plane-to-plane transitioning.

## Teaser

Scalable electrostatic rollers have rapid speed, superior load-to-weight ratio, and multimodal locomotion capabilities.

## INTRODUCTION

Crawling and climbing are two key forms of terrestrial animal locomotion (*1*, *2*). Various locomotive creatures such as geckos (*3*) and snails (*4*) are capable of versatile crawling and climbing so that they can survive in unknown and unstructured living environments. Mobile robots that combine crawling and climbing can greatly increase their moving ranges and dimensions and enhance their capabilities in completing tasks in complex working conditions. To this end, various solutions, in the form of wheeled, legged, or tracked locomotion mechanisms, have been developed including (i) traditional electromechanical actuation combined with magnetic adhesion (*5*), suction adhesion (*6*), gecko-inspired adhesion (GA) (*7*, *8*), and electroadhesion (EA) (*9*–*12*); (ii) pneumatic actuation with SA (*13*, *14*) and EA (*15*); (iii) shape memory alloy actuation with EA (*16*); (iv) dielectric elastomer actuation (DEA) with EA (*17*); (v) piezoelectric actuation with EA (*18*); (vi) magnetic actuation with GA (*19*); (vii) liquid crystal elastomer actuation with EA (*20*); and (viii) electrostatic actuation with EA (*21*, *22*). These climbing approaches usually separate actuation and adhesion, thus resulting in cumbersome structures or complex control strategies, and their moving speeds [in terms of body length per second (BL/s)] are relatively slow.

Rolling is a simple and efficient locomotion mechanism on relatively flat surfaces compared to legged and tracked locomotion. Some animals such as pangolins (*23*) and golden wheel spiders (*24*) may use their whole-body rolling for defending and rapid escaping. Humans often use wheel rolling for fast transportation (*25*).

There have been several electrically driven rollers for versatile crawling such as EA rolling (*26*), multisegment DEA-based rolling (*27*), liquid metal–based rolling (*28*), and electrohydrodynamic-based rolling (*29*). These robots require either complex structures with sophisticated control strategies or expensive fabrication, and they did not demonstrate successful climbing.

**Fig. 1.**
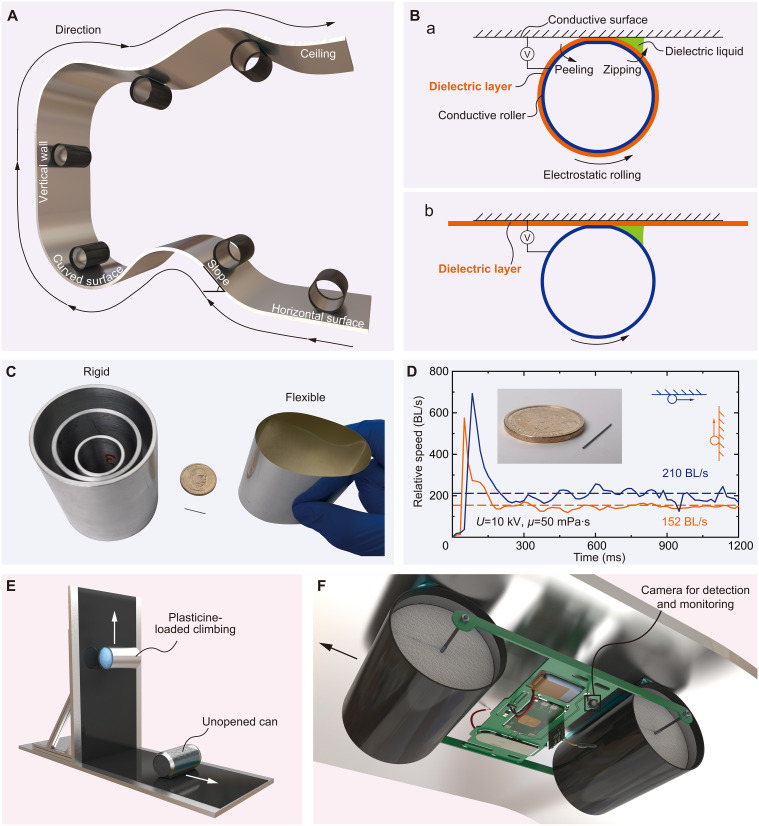
Rapid, versatile moving by LAER robots. (**A**) Schematic diagram of rapid crawling and climbing of LAER rollers on conductive surfaces with various curvatures. (**B**) Schematic diagram of LAER structures. (**C**) Prototypes of centimeter-scale (diameters ranging from 65.0 to 0.5 mm) and submillimeter-scale rigid (mass of 0.015 g) and centimeter-scale flexible LAER rollers (diameter of 53.0 mm and mass of 3.620 g). (**D**) Climbing speed-time curves for the submillimeter-scale rigid LAER roller. (**E**) Climbing with a load-to-weight ratio of ~16 for a flexible LAER roller and crawling with a load-to-weight ratio of ~121 for an unopened can. (**F**) An untethered LAER robot performing environmental detection and monitoring whilst climbing invertedly on conductive surfaces.

Here, we present a liquid-amplified electrostatic rolling (LAER) actuator, elegantly combining parallel EA and electrostatic actuation into an adhesion-actuation integrated structure, for versatile moving (see Fig. 1A) on horizontal surfaces (movies S1 and S2), curved surfaces (movie S3), slopes (movie S4), vertical walls (movie S4), and ceilings (movie S4), when a voltage is applied. The LAER mechanism can be realized by either an insulated conductive roller moving on bare conductive surfaces [see Fig. 1B (a)] or a bare conductive roller moving on insulated conductive surfaces [see Fig. 1B (b)], guided by a tiny volume of dielectric liquid on one side of the roller, that produces asymmetric electrostatic force distributions for crawling and climbing. The LAER roller can be made of rigid or flexible structures and the roller is scalable (see Fig. 1C). The small-scale rigid tethered LAER roller (diameter of 0.5 mm and mass of 0.015 g) exhibited a rapid vertical climbing speed of ~152 BL/s and inverted climbing speed of ~210 BL/s, as shown in Fig. 1D and movie S5, and the large-scale flexible tethered LAER roller (diameter of 66.2 mm) demonstrated superior load-to-weight ratios such as ~121 for crawling and ~16 for vertical climbing, as shown in Fig. 1E and movie S7. We combined experimental data and scaling analysis to obtain the governing parameter group for crawling to unravel the dynamic behavior of LAER rollers.

Liquid regulating modules were designed and embedded into LAER structures to exhibit controlled moving (see movies S8 and S9). Two modules connected in parallel produced a robot with controlled turning and in series produced a robot with controlled reciprocating moving. We also developed a single-wheeled (see movie S10) and a two-wheeled untethered LAER robots (see movie S12) with onboard power supply. To demonstrate the potential application of LAER robots, an electrically controllable LAER linear actuator (see movie S11) and a camera incorporated untethered LAER robot for environmental detection and monitoring were demonstrated. The two-wheeled camera equipped untethered LAER robot (see Fig. 1F) can be used to crawl and climb both vertically and invertedly (see movies S12 to S14) on various conductive materials.

## RESULTS

### LAER mechanism

The electrostatic pressure, $P\propto \epsilon E^{2}$ (*30*, *31*), is generated between the LAER roller and the conductive surface (see fig. S1), when a potential difference is applied, where E is the electric field strength that increases with increasing voltage and ϵ is the permittivity of the dielectric. The electric field strength in liquid or air cannot continue to increase when there is a dielectric breakdown and is assumed that it remains as the breakdown strength $E^{\text{breakdown}}$ (*32*). Placing a tiny volume of dielectric liquid on one side of the roller results in an asymmetric distribution of electrostatic forces (see fig. S2B), due to the fact that the permittivity and breakdown strength of the dielectric liquid are greater than air (*32*). The electrostatic force components, $F^{//}$ and $F^{⊥}$ , in direction tangential and normal to conductive surfaces, provide electrostatic actuation and adhesion, respectively, for climbing. On the liquid side, the related conductive roller area is continuously zipped to the conductive surface, whereas, on the air side, the related roller area is constantly departed from the conductive surface, thus resulting in a rapid and versatile electrostatic rolling.

Maintaining the dielectric droplet on one side is crucial for the continuous and stable rolling of LAER rollers. The electric field strength of the liquid side is much higher than the air side and dielectric liquid is prone to move toward places where the field strength is higher due to dielectrophoretic forces (*32*–*34*). Continuous voltage application then retains the droplet on one side of the LAER roller, leading to continuous and stable rolling.

### Dynamic behavior of rigid LAER rollers

**Fig. 2.**
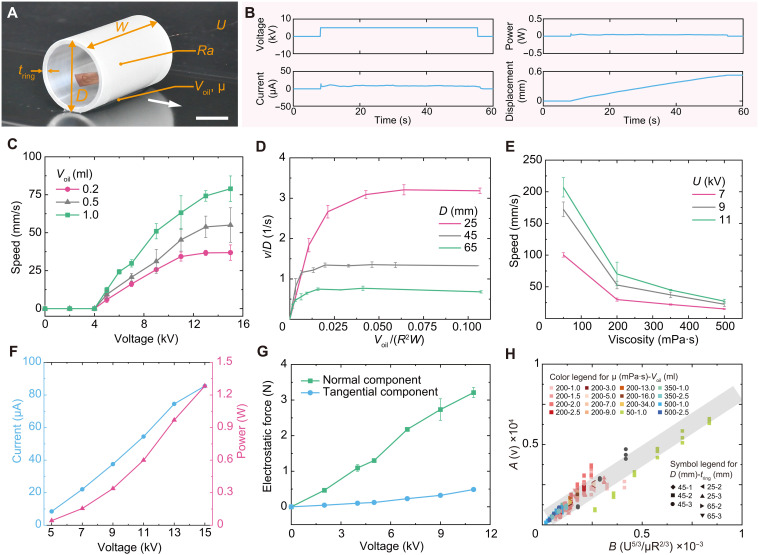
Dynamic behavior of centimeter-scale rigid tethered LAER rollers. (**A**) Experimental snapshot of a centimeter-scale rigid LAER roller in speed characterization experiments. (**B**) Voltage, current, electrical power traces, and displacement during an experiment for U=5.00 kV. (**C**) The speed with respect to the voltage for different dielectric liquid volumes. (**D**) The speed as a function of the dielectric liquid volume for different roller diameters. (**E**) The speed in term of the dielectric liquid viscosities for different applied voltages. (**F**) Effect of the voltage on the average current and power during crawling. (**G**) Influence of the voltage on the electrostatic forces. (**H**) Scaling relations of the speed and the governing parameter group regarding experimental data in the voltage-dominated regime. *A* and *B* in axes labels represent the product terms in equation S33. Scale bar, 20 mm.

There are two locomotive stages, i.e., the acceleration stage and the stable moving stage (see Fig. 1D and fig. S8, D to F), associated with rigid LAER rollers (see Figs. 1C and 2A). By considering the Newton’s second law of motion and the angular momentum theorem, the dynamic governing equations for rigid LAER rollers were developed (see section S1.1) to derive the starting acceleration (see section S1.1.1). We then used the Poiseuille flow model (*35*–*37*) to obtain the dielectric liquid viscous resistance. The approximate expression for the stable speed ( v ) (see section S1.1.2) can then be obtained when the viscous resistance is balanced by the net driving force, $F^{//}$.

Here, a customized experimental setup was adopted to characterize the crawling speed of centimeter-scale rigid LAER rollers (see Fig. 2A), considering the effect of the applied voltage ( U ), the dielectric liquid volume ( $V_{\text{oil}}$ ) and viscosity (μ), the roller diameter ( D ), the roller thickness ( $t_{\text{ring}}$ ), and the roller surface texture. The displacement-time curves (see fig. S7) of centimeter-scale rigid rollers during the stable moving stage were recorded by a displacement sensor, and the stable speeds were obtained by a linear fitting. We adopted the aluminum-based bare conductive roller [see Fig. 2B (b)] for all of the characterization experiments. The default settings were U=9.00 kV, $V_{\text{oil}}=1.00$ ml, μ = 200 mPa·s, D=45.0 mm, and $t_{\text{ring}}=2.0$ mm.

Three LAER rollers with different surface textures, designated as smooth, medium, and rough (for machining processes see Materials and Methods for details), were prepared. Smooth LAER rollers were able to move forward in a pure rolling mode when the voltage was lower. When the voltage was higher, the friction provided by the roller-base interface was not sufficient to support higher speeds, and sliding (or turning) occurred. The rough LAER rollers did not move and the liquid dielectric flowed from the liquid side to the air side through the surface grooves until symmetrically distributed, when an electric field was applied. The “medium” LAER rollers in the applied voltage range (5 to 15 kV) were able to move forward in a straight trajectory.

Therefore, the medium roller surface texture was used for the later characterization experiments of large-scale rigid rollers. Figure 2B depicts the applied voltage, current, power, and displacement of the LAER roller as a function of time when the applied voltage was 5.00 kV. The average current was ~8.5 μA, and the resulting average power consumption was ~42.4 mW.

Figure 2C illustrates the speed varying with the applied voltage for different dielectric liquid volumes. When the voltage was over a threshold, 5 kV, the LAER roller could roll forward continuously and stably, and the moving speed increased with increasing the applied voltage. When the voltage was below the threshold, the roller remained stationary or rolled for a short distance and then stopped moving, during which the asymmetric distribution of the dielectric liquid gradually disappeared. In the applied voltage range of 11 to 15 kV for the liquid volume of 0.20 ml and in the range of 13 to 15 kV for the liquid volume of 0.50 ml, the moving speeds were less affected by the applied voltage, which maybe due to the fact that the voltage was high enough to make the whole dielectric liquid breakdown. Therefore, we defined this regime as the volume-dominated regime, where the speed is determined by liquid volume and nearly independent of voltage when the applied voltage is high enough.

The relative speeds as a function of the liquid volume for different roller diameters are shown in Fig. 2D. The speed first increased with volume and then remained almost constant. This maybe because that there is a limit to the liquid volume that can be retained on the liquid side by the action of the electric field. We defined this regime as the voltage-dominated regime, where the speed is almost independent of the liquid volume and depends on the voltage when the liquid volume is large enough. Between the two regimes is a transition regime, where both the volume and the applied voltage had a notable influence on the speed. In addition, the angular speed decreased with increasing roller diameter when the liquid volume was given. The effect of dielectric liquid viscosity on the speed is plotted in Fig. 2E. For a given applied voltage, the speed decreased with increasing liquid viscosity, due to the fact that the greater the liquid viscosity, the greater the viscous resistance.

Figure 2F depicts the average current and power as a function of the applied voltage for crawling. Both the average current and power increased with increasing voltage. Two customized experimental setup (see the insets of figs. S3A and S4A) were adopted to measure the electrostatic force components, $F^{//}$ and $F^{⊥}$ , in direction tangential and normal to conductive surfaces, respectively. Figure 2G illustrates the tangential and normal electrostatic force components as a function of voltage. Both electrostatic force components increased with increasing voltage. The normal component of electrostatic force was ~10 times larger than the tangential component.

A scaling analysis was performed to obtain the parameter group that governs the locomotion behavior of rigid LAER rollers (see section S1.3). The crawling speeds in the volume- and voltage-dominated regimes can be determined as $v\propto V_{\text{oil}}/\left(\mu R\right)$ and $v\propto U^{\frac{5}{3}}/\left(\mu R^{\frac{2}{3}}\right)$ , respectively, where R is the roller radius. The scaling relation of equation S33 in the voltage-dominated regime was reasonably consistent with the experimental results, as demonstrated in Fig. 2H.

### LAER structural design further considerations

The estimation of moving (i.e., crawling and climbing) performance of LAER rollers, including the moving speed and the load-carrying capacity, is dependent on two important factors, the effective actuation and adhesion between the roller and the base, which are related to the provided electrostatic forces. During crawling, the roller-base adhesion is always effective, and the actuation performance is mainly determined by $F^{//}$ . During vertical climbing, the roller-base adhesion is ensured by $F^{⊥}$ , and the effective actuation can be realized only if $F^{//}-\mathrm{mg}>0$ . During inverted climbing, the roller-base adhesion can be realized only if $F^{⊥}-\mathrm{mg}>0$ , and the effective actuation is provided by $F^{//}$.

Because the normal electrostatic force $F^{⊥}$ was notably larger than the tangential electrostatic force $F^{//}$ , the vertical climbing is the most challenging among the three moving modes. An important parameter for the moving performance of LAER rollers is defined as $\gamma =F^{//}/\mathrm{mg}$ . The greater the γ , the better the LAER moving performance, and the necessary condition for vertical climbing is γ>1 . From equations S26 and S28 of the scaling analysis, we obtained that $F^{//}\propto R^{-\frac{1}{3}}$ and $F^{//}\propto R^{0}$ in volume- and voltage-dominated regimes, respectively, when varying only the roller radius. Due to mg∝R for thin-walled rollers, $\gamma \propto R^{-\frac{4}{3}}$ and $\gamma \propto R^{-1}$ can be obtained in volume- and voltage-dominated regimes, respectively. Therefore, reducing the roller diameter may be an efficient way to improve the moving performance of LAER rollers.

### Submillimeter-scale rigid LAER rollers

**Fig. 3.**
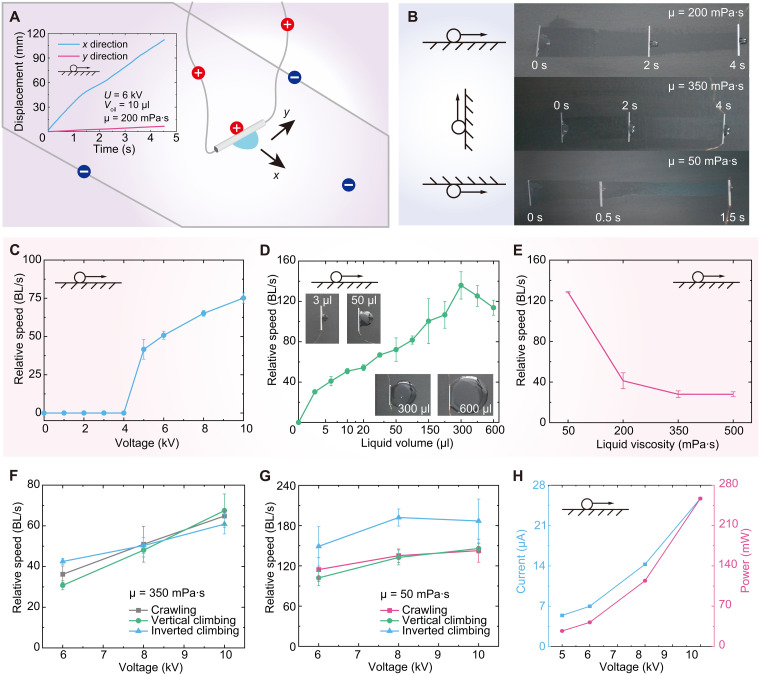
Dynamic behavior of submillimeter-scale rigid tethered LAER rollers. (**A**) Schematic diagram of speed characterization experiments for submillimeter-scale rollers. The inset is the displacement-time curves obtained from the experimental video by the software Tracker. (**B**) Experimental snapshots of crawling and vertical and inverted climbing of the submillimeter-scale roller for different liquid viscosities. (**C**) Effect of the applied voltage on the crawling speed. (**D**) Effect of the liquid volume on the crawling speed. (**E**) Effect of the liquid viscosity on the crawling speed. (**F**) Crawling and vertical and inverted climbing speeds in term of the voltage for liquid viscosity of 350 mPa·s. (**G**) Crawling and vertical and inverted climbing speeds in term of the voltage for liquid viscosity of 50 mPa·s. (**H**) Effect of the voltage on the average current and power during crawling.

Here, a stainless steel LAER roller with a diameter of ~0.5 mm and a weight of ~0.015 g was fabricated to characterize its crawling and vertical and inverted climbing speeds. The submillimeter-scale roller was connected to the power supply by passing a 0.025 mm diameter of the suspended copper wire through the roller (see Fig. 3A). The displacement in the inset of Fig. 3A illustrates that the roller moved continuously at a constant speed, and the trajectory was approximately straight. Figure 3B shows experimental snapshots of the crawling and vertical and inverted climbing of the submillimeter-scale roller with different liquid viscosities. The default settings were U=6.00 kV, $V_{\text{oil}}=10$ μl, and μ = 200 mPa·s.

As the roller diameter was reduced to submillimeter level, the edge effect (electric field distribution along the edge of the small roller) cannot be ignored.

The dielectric liquid was confined to the middle of the roller under the action of applied electric field, as shown in Fig. 3B. Figure 3C shows the relative crawling speed (BL/s, here, we define BL as the outer diameter of the LAER roller) as a function of the applied voltage. The speed increased as the voltage increased when the voltage was over the threshold, 5.00 kV. The influence of liquid volume on the crawling speed of the submillimeter-scale roller is depicted in Fig. 3D. The speed first increased to a maximum speed at 300 μl and then decreased as the liquid volume increased. Experimental snapshots of different liquid volumes are shown in the insets of Fig. 3D. The crawling speed as a function of liquid viscosity is illustrated in Fig. 3E, which decreased rapidly and then slowly with liquid viscosity.

The crawling and vertical and inverted climbing speeds versus voltage for the high liquid viscosity of 350 mPa·s are illustrated in Fig. 3F. These speeds increased with increasing voltage and had little difference for a given voltage. Figure 3G illustrates the crawling and vertical and inverted climbing speeds regarding voltage for the low liquid viscosity of 50 mPa·s. Unlike the high viscosity case, the inverted climbing speed was faster than the crawling and vertical climbing speeds, which may be due to the fact that the low viscosity droplet had a greater distribution angle (see fig. S1) on ceilings under gravity. The observed maximum vertical and inverted climbing speeds in Fig. 3G are ~152 and ~210 BL/s, respectively, under U=10.00 kV, $V_{\text{oil}}=10$ μl, and μ = 50 mPa·s. These two maximum speed values represent the quickest vertical and inverted climbing speeds among all published climbing robots, as compared and shown in Table 1. Movie S6 presents a slow-motion (0.05×) playback of the crawling process of the submillimeter-scale rigid LAER roller, captured via a high-speed camera. See section S1.6 for details. In addition, these maximum speeds can be further increased by increasing the applied voltage and liquid volume or decreasing the liquid viscosity. Both average current and power of submillimeter-scale rigid roller during crawling increased with increasing the applied voltage, as shown in Fig. 3H.

**Table 1.** Comparison of major crawling and climbing robots. Note: Our work and crawling and climbing robots are categorized and listed. NA, not available.

Ref.	Weight (mg)^*^	Structure^†^	Mechanism^‡^	Forward crawling		Vertical climbing		Other climbing modes	
				Speed (BL/s)	Load-to-weight ratio	Speed (BL/s)	Load-to-weight ratio	Inverted climbing speed (BL/s)	Circular climbing
Tethered LAER roller	1.50E+01 (T)	1 DoF + rolling	LAER	162.40	120.62	152.20	16.47	210.00	√
(*41*)	5.00E+03 (T)	1 DoF + multilegged	Pneumatic actuator	~0.08	>400.00	NA	NA	NA	NA
(*42*)	1.72E+02 (T)	1 DoF + 2-legged	Electromagnetic actuator	70.00	~0.20	NA	NA	NA	NA
(*43*)	2.40E+01 (T)	1 DoF + 2-legged	Piezoelectric actuator	20.00	~6.34	NA	NA	NA	NA
(*44*)	5.80E+01 (T)	1 DoF + 2-legged	Piezoelectric actuator	42.80	~8.03	NA	NA	NA	NA
(*45*)	8.80E+02 (T)	6 DoF + rolling	Multisegment DEA	0.95	NA	NA	NA	NA	NA
(*20*)	2.00E+02–3.00E+03 (T)	5 DoF + 2-legged	Electrothermal actuator + EA	NA	NA	~0.02	NA	NA	√
(*46*)	4.34E+04 (T)	2 DoF + 2-legged	Pneumatic actuator + EA	0.12	69.00	~0.05	NA	NA	NA
(*15*)	2.57E+03 (T)	3 DoF + 2-legged	Pneumatic actuator + EA	0.08	0.47	0.08	0.47	0.08	√
(*17*)	NA	1 DoF + 2-legged	DEA + EA	1.04	NA	0.75	NA	NA	NA
(*18*)	1.48E+03 (T)	8 DoF + 4-legged	Piezoelectric actuator + EA	~3.11	NA	~0.03	NA	~0.10	√
(*16*)	<5.00E+04 (T)	2 DoF + 4-legged	Shape memory alloy + EA	~0.01	NA	~0.01	NA	NA	NA
(*13*)	~3.33E+04 (T)	3 DoF + 2-legged	Pneumatic actuator + PA	0.11	~15.00	~0.08	~0.60	NA	NA
(*14*)	<4.00E+04 (T)	1 DoF + 2-legged	Pneumatic actuator + PA	NA	>8.75	~0.03 (loaded)	>5.00	NA	NA
Single-wheeled untethered LAER robot	3.04E+04 (U)	1 DoF + rolling	LAER	6.62	NA	NA	NA	NA	NA
Two-wheeled untethered LAER robot	4.76E+04 (U)	2 DoF + rolling	LAER	0.24	NA	0.04	NA	0.10	NA
(*47*)	~2.00E+00 (U)	1 DoF + rolling	Magneto-elastomer actuator	~57.57	NA	NA	NA	NA	NA
(*48*)	~8.00E+00 (U)	1 DoF + rolling	Light-driven hydrogel actuator	50.00	NA	NA	NA	NA	NA
(*49*)	~3.90E+01 (U)	1 DoF + multilegged	Magneto-elastomer actuator	~2.82	>100.00	NA	NA	NA	NA
(*50*)	2.10E+02 (U)	1 DoF + rolling	Light-driven actuator	<35.95	NA	NA	NA	NA	NA
(*28*)	1.13E+04 (U)	1 DoF + rolling	Liquid metal–based actuator	1.25	NA	NA	NA	NA	NA
(*12*)	~2.00E+06 (U)	1 DoF + tracked	Electric motor + EA	NA	NA	~0.14 (80°)	NA	NA	×
(*51*)	5.00E+04–6.00E+04 (U)	2 DoF + 2-wheeled	Electric motor + PA	NA	NA	NA	NA	NA	√
(*52*)	~1.33E+06 (U)	5 DoF + 4-wheeled	Electric motor + PA	~13.33	~30.00	~1.04	NA	NA	NA
(*5*)	~8.00E+06 (U)	12 DoF + 4-legged	Electric motor + MA	NA	NA	2.12	~0.25	1.51	√
(*19*)	NA (U)	1 DoF + 2-legged	Magneto-elastomer actuator + GA	NA	~20.00	NA	~20.00	NA	√
(*53*)	3.70E+05 (U)	12 DoF + 4-legged	Electric motor + GA	0.72	NA	0.12	NA	NA	NA
(*54*)	1.50E+04 (U)	1 DoF + 6-legged	Electric motor + GA	NA	NA	2.40 (near vertical)	NA	NA	NA
(*55*)	9.00E+03 (U)	1 DoF + 2-legged	Electric motor + GA	NA	NA	0.60	>100.00	NA	NA

* “T” and “U” refer to “tethered” and “untethered,” respectively.

† “DoF” denotes the actuation degree of freedom of robots.

‡ “DEA” refers to dielectric elastomer actuator; “EA” denotes electroadhesion; “PA” denotes negative-pressure adhesion; “MA” denotes magnetic adhesion; and “GA” denotes gecko-inspired adhesion.

### Load-carrying and controlled moving of tethered multimodal LAER robots

**Fig. 4.**
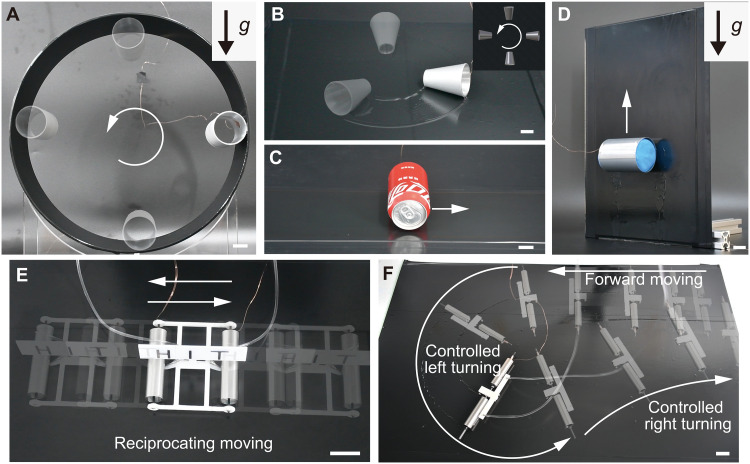
Load-carrying and controlled moving of tethered multimodal LAER robots. (**A**) Experimental snapshots of circular climbing of a centimeter-scale rigid LAER roller. (**B**) Experimental snapshots of circular moving of a centimeter-scale tapered LAER roller. (**C**) Experimental snapshots of forward moving of an unopened readily available Coca-Cola can (330 ml). (**D**) Experimental snapshots of vertical climbing of flexible LAER rollers with a load to weight ratio of ~16. (**E**) Reciprocating moving and controlled stops of the serial LAER robot with liquid regulating modules. (**F**) Controlled turning of the parallel LAER robot with liquid regulating modules. The white arrows indicate the moving direction. Scale bars, 20 mm.

The circular climbing was achieved by the large-scale rigid LAER roller in Fig. 4A and by the submillimeter-scale rigid LAER roller in fig. S5C. We fabricated a tapered rigid LAER roller, of which one end was D=45.0 mm and the other end was D=25.0 mm. The circular moving was achieved by the tapered roller with $V_{\text{oil}}=1.50$ ml, μ = 200 mPa·s, and U=8.00 kV, as shown in Fig. 4B. The circular trajectory can be adjusted by the dimensions of the tapered structure. Figure 4C demonstrates forward moving of a large-scale LAER roller by directly using an unopened readily available Coca-Cola can (330 ml) at a speed of 0.12 BL/s under U=4.75 kV, μ = 20 mPa·s, and $V_{\text{oil}}=0.60$ ml (see movie S7).

We also fabricated flexible thin-walled LAER rollers (see Fig. 4D) with a thickness of ~0.1 mm, which can be used to provide greater adhesive areas (*38*) and thus greater electroadhesive forces. Figure S9 illustrates the effect of the applied voltage, liquid volume, and viscosity on the crawling speed of flexible LAER rollers. It can be observed that the speed increased with increasing voltage, liquid volume, and viscosity. The dielectric liquid viscosity had opposite effects on speeds of flexible LAER rollers compared to rigid ones, which may be due to the deformation of the flexible rollers. In Fig. 4D, vertical climbing with a load to weight ratio of ~16 was realized by installing plasticine embedded with 5-mm-diameter steel balls inside LAER flexible rollers (see movie S7). Crawling with a load to weight ratio of ~121 was achieved by filling a can with liquid metal (see fig. S6C and movie S7).

To achieve controlled moving of the LAER structure, serial and parallel LAER robots were designed and developed in figs. S16 and S17. The controlled moving can be achieved by adjusting the speed difference between different LAER rollers. In addition to applied voltage (see Fig. 2C), dielectric liquid volume (see Fig. 2D) also had a notable impact on the roller speed. Liquid regulating modules were introduced into serial and parallel LAER structures to regulate the volume of the dielectric liquid.

Figure 4E and movie S8 show the reciprocating moving and controlled short stops of the serial LAER robot with liquid regulating modules. The detailed illustration of the serial LAER robot can be found in section S1.9 and fig. S16A. By providing the voltage and dielectric liquid only to the right (or left) roller, the serial LAER robot can move to the left (or right). In addition, the serial LAER robot could be started and stopped by controlling the voltage switch, and the response was relatively fast. The displacement-time curve of the reciprocating moving and short stops of the serial LAER robot is shown in fig. S16B.

Controlled turning of the parallel LAER robot is shown in Fig. 4F and movie S9. The detailed illustration of the parallel LAER robot can be found in section S1.10 and fig. S17. The identical voltage was applied to both rollers. Two independent liquid regulating modules allowed the independent adjustment of the dielectric liquid volume (or moving speed) for each LAER roller. The controlled turning was achieved by introducing a speed difference between different LAER rollers.

### LAER robots for linear actuators and environmental monitoring

**Fig. 5.**
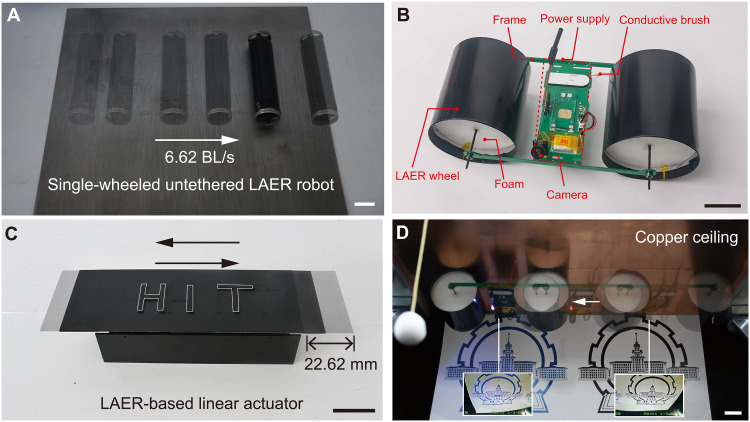
LAER structures for linear actuators and environmental monitoring. (**A**) Rapid moving of the single-wheeled untethered LAER robot on the stainless steel. (**B**) Prototype of the two-wheeled untethered LAER robot for environmental monitoring and detection. (**C**) Reciprocating motion of the LAER-based linear actuator with a stroke length of 22.62 mm. (**D**) Snapshots of the two-wheeled untethered LAER robot climbing on a copper ceiling while performing environmental monitoring. The arrows indicate the moving direction. Scale bars, 20 mm.

A single-wheeled untethered LAER robot with diameter of 25 mm was developed in fig. S18. The detailed illustration can be found in section S1.11. Figure 5A and movie S10 shows that the single-wheeled untethered LAER robot moving at an average speed of 6.62 BL/s on stainless steel surfaces. Figure S18 (B and C) shows the displacement-time and speed-time curves, respectively. It can be seen that the maximum instantaneous speed was 286.90 mm/s (11.48 BL/s). The speed of the single-wheeled untethered LAER robot could be further improved if the voltage amplifier is smaller in size and can output higher voltages.

To showcase the applicability of the LAER mechanism, we designed and developed a two-wheeled untethered LAER robot for environmental monitoring and detection. The mechanical and electronic design of the two-wheeled untethered LAER robot can be seen in Fig. 5B and fig. S19, respectively, and all components of the untethered robot are listed in table S1.

The two-wheeled untethered LAER robot developed in this study adopted the structural form in Fig. 1B (a), allowing it to climb on bare conductive surfaces, such as some hull surfaces, oil storage tank surfaces, and ceilings of manufacturing plants. To reduce the weight of the wheels, the stiffness-enhanced rollers were fabricated by filling the flexible roller with cylindrical stiff foams. The robot was made of two stiffness-enhanced wheels (diameter of 66.2 mm and width of 65.0 mm) connected with one pole of a high-voltage converter (EMCO AG60P-5, XP Power Co. Ltd.) that was powered by a 3.7-V Li-Po battery (100 mAh). The elastic conductive brush connected to the other pole of the high-voltage converter could ensure stable contact with bare conductive surfaces to form a potential difference between the two wheels and the conductive surfaces. A voltage booster (DM31-10W05S, Chengdu Bary Technology Co. Ltd.) was used to increase 3.7 to 5 V so that the high-voltage converter can output 6 kV. The robot was demonstrated to crawl and climb (both vertically and invertedly) on different conducting materials such as copper, aluminum, and stainless steel (see movies S12 to S14). A wireless camera (ZENCHANSI 800TVL) was mounted on the robot for environmental monitoring uses. Figure 5D demonstrates that the LAER robot climbing on a copper ceiling while performing environmental monitoring tasks (see the insets).

In addition, we designed and developed a LAER-based linear actuator in Fig. 5C. The detailed illustration and design are illustrated in section S1.13 and fig. S20, respectively. The two LAER rollers were hinged but can rotate, and the conductive plate above both rollers moved forward under an electric field. Using the control method similar to that of the serial LAER robot, reciprocating motion of the LAER-based linear actuator was achieved (see movie S11). The demonstrated stroke length was 22.62 mm, and it is convenient to increase the stroke length by increasing the number of serial rollers and the length of the conductive plate.

## DISCUSSION

In this work, we have designed, fabricated, and characterized a LAER structure for rapid, versatile robotic locomotion. Current climbing robots have been separating the actuation and adhesion function. Here, we integrate the actuation and adhesion in a single-degree-of-freedom structure by adding a tiny volume of dielectric liquid to one side of the LAER roller, thus producing asymmetric electrostatic forces, where the normal force component is used for adhesion and the tangential component is used for rolling. This streamlined, elegant design can reduce robot structural complexity and mass, representing a promising climbing mechanism that can be easily scalable and super-lightweight, which can be seen in Fig. 1C, where the diameters of the rigid LAER structure can range from 0.5 mm (0.015 g) to 65.0 mm (114.110 g).

LAER structures are easy to make and can be made of both rigid and flexible materials and structures. We have conducted a scaling analysis, combining theoretical considerations and experimental data, to obtain the speed governing parameter group for understanding the dynamic behavior of LAER actuators and their structural design.

The LAER mechanism can be used to have multiple locomotive functions (Fig. 4 and figs. S5 and S6) including forward moving (movie S1), turning (movie S2, by local texturing the roller), circular moving (movie S2, by tapering the roller), circular climbing (movie S3), vertical climbing (movie S4), inverted climbing (movie S4), and plane-to-plane transitioning (movie S4). To the best of the author’s knowledge, the LAER design has produced a lightweight, submillimeter-scale rigid tethered roller that had the quickest vertical and inverted climbing relative speed, ~152 and ~210 BL/s, respectively, and a centimeter-scale flexible tethered roller that exhibited a superior crawling load-to-weight ratio of ~121, compared to the existing crawling and climbing robots (see Table 1).

We have designed and developed a single-wheeled (speed of 6.62 BL/s) and a two-wheeled untethered LAER robot with onboard power supply (see Fig. 5, A and B). The two-wheeled untethered LAER robot was mounted with a wireless camera and can be used to crawl and climb both vertically and invertedly on different conducting materials for environmental detection and monitoring tasks (see Fig. 5D and movies S12 to S14). It should be noted that other wireless sensors such as temperature/humidity sensors for environmental temperature/humidity sensing can also be added for multifunctional environmental detection and monitoring tasks in various locations.

Despite the aforementioned advantages of the LAER mechanism, the moving performance of LAER robots is limited by its liquid depletion. To this end, we designed a liquid regulating device that can be used to replenish and reduce the dielectric liquid needed for controlled and prolonged moving (Fig. 4, E and F). To demonstrate the usability of the LAER mechanism apart from detection and monitoring, we combined the liquid regulating module with two LAER rollers to produce a linear actuator with controlled reciprocating motions, and this electrically controllable linear actuator should be used for linear rails, conveyors, among others.

Although we have shown that LAER robots can be used on different materials such as copper, aluminum, stainless steel, and iron, their moving performance can be notably affected on oxidized, dirty, rusty surfaces, wet surfaces with water droplets, and bumped surfaces with small obstacles (see movie S15). It should be noted that the moving performance of LAER robots can be influenced if the surface is too rough or not flat or not neat enough, and this may limit the wider application of the LAER mechanism and requires further improvements. It can be seen that the moving speed of the developed untethered LAER robots was notably slower than the tethered versions. This is due to the fact that the moving speed is directly related to the roller diameter and mass. In the future, high-*k* dielectrics (*39*) can be incorporated into LAER robots to further reduce the required voltage and robot weight. Advanced micro/nanomanufacturing methods can also be used to smaller the roller size and weight, thus increasing the moving speed of untethered LAER robots and also promoting the developed linear actuators for microelectromechanical actuation systems (*40*).

## MATERIALS AND METHODS

### LAER actuator materials and fabrication methods

Centimeter-scale rigid bare rollers were made of aluminum and manufactured by Computer Numerical Control (CNC) machining. All roller widths were 75.0 mm. The smooth surface textures on the rollers were CNC-machined surfaces without postprocessing. The medium surface textures on the rollers were obtained by applying scratches along the direction of roller width using a stainless steel wire brush with a wire diameter of 0.25 mm. The rough surface textures on the rollers were mesh knurled textures with a spacing of ~1.0 mm and a depth of ~0.2 mm.

Centimeter-scale flexible bare rollers were made by an aluminum stamping process or obtained by wire electrical discharge machining (WEDM) of commercially available aluminum cans, and the surfaces of all flexible rollers were untreated. The thickness and width of all the centimeter-scale flexible rollers were ~0.1 and ~65.0 mm, respectively.

Submillimeter-scale rigid bare rollers were obtained by WEDM of commercially available stainless steel capillary rods. The submillimeter-scale rigid rollers had an outer diameter of ~0.5 mm, an inner diameter of ~0.3 mm, a width of ~15.0 mm, and a weight of ~0.015 g. The surfaces of all submillimeter-scale rigid rollers were sanded with a 360 grit sandpaper. Conductive surfaces were made from a ~2.0-mm-thick stainless steel plate. Two layers of polyvinyl chloride tapes (thickness of ~0.18 mm, SUPER 33+, 3M Co.) were laminated together for the insulation of the conductive surfaces. Silicone oils (PMX-200, Shanghai Aladdin Biochemical Technology Co. Ltd.) with different viscosities were used as the dielectric liquid.

### Speed characterization

A laser displacement sensor (see the inset of fig. S7A, FLR-50, Fuwei Electronics Inc.) was used to measure displacements over time of centimeter-scale LAER rollers. It should be noted that the initial acceleration stage (displacement of roughly 50 mm) was not considered and recorded for centimeter-scale rollers. Then, the roller speeds were obtained as the slopes of linear fittings for the stable linear segment of the displacement-time curves (see figs. S7 and S8). The high-voltage power supply (72030PA, Boher HV Inc.) was used to provide the applied voltage. The specific volume of dielectric liquid was added to the middle of the roller liquid side, by a pipette (1000 to 5000 μl, JoanLab, CHINA) before applying the voltage in all experiments. Each test was repeated three times and the average (denoted as points) plus one SD (denoted as error bars) was plotted.

For submillimeter-scale LAER rollers and the untethered robot, a camera (D7500, Nikon Inc.) was used to record their movements at a frame rate of 59.94 frames per second (fps) and a resolution of 1920 × 1080 pixels. Their speeds were measured by analyzing the frames of the obtained videos. To better locate the roller position in the frame, we used a black background to stand out the silvery white submillimeter-scale rollers. The position of submillimeter-scale rollers in each frame can be obtained by tracking their feature points with software “Tracker.” Here, all reported speeds were steady-state results. In addition, the speeds of submillimeter-scale rollers over time can also be calculated by software Tracker. The high-voltage power supply (72030PA, Boher HV Inc.) was used to provide the applied voltage. The pipettes of 0.5 to 10 μl and 10 to 2000 μl (DragonLab, CHINA) were used to add a specific volume of dielectric liquid to the middle of the roller liquid side. The submillimeter-scale rigid roller was connected to the power supply by the copper wire (CU5162, Advent International Inc.). Each speed test was repeated three times.

### Rigid LAER actuator force test

A load cell (Richmond Industries Inc.) was used to characterize the tangential electrostatic forces (see the insets of fig. S3A). To measure the net driving force, the load cell was connected with a three-dimensional (3D) printed insulating rod that was directed to the center of the roller and gently attached to the roller surface. Through the force analysis for the roller, it can be seen that the load cell can directly measure the net driving force (see section S1.2.2). The force-time curve (see fig. S3A) was obtained. A high-voltage power supply (72030PA, Boher HV Inc.) was used to provide the applied voltage. The pipette of 1000 to 5000 μl (JoanLab, CHINA) was used to add a specific volume of dielectric liquid to the middle of the roller liquid side. Each force test was repeated three times.

The normal electrostatic forces (see the inset of fig. S4A) were also characterized. The roller and the load cell were connected together by a 3D printed clamp and fixed to a linear stage (X-LSQ450A-E01-KX14C, Zaber Technologies Inc.). At the beginning, a preload force was applied to the roller to ensure complete contact with the base. Then, a voltage was applied, and a dielectric liquid was added to both sides of the roller, and the roller was lift from the base at a speed of 10 μm/s by the linear stage. The force-time curve (see fig. S4A) was obtained.

### Liquid regulating module

The liquid regulating module comprised a needle, a soft tube, and a syringe. The volume of dielectric liquid near the needles can be adjusted by controlling the syringe. The inner diameter of the needles was 0.8 mm. The flexible tube, with inner diameter of 1.01 mm (TYGON AAD04103, SAINT-GOBAIN PPL CORP.), was used to connect the needles and the syringes. The outlet ports (i.e., the needles) of two independent liquid regulating modules were mounted on 3D printed frames.
