# Supplementary material for: Rapid moving by liquid-amplified electrostatic rolling
Source: Sci Adv. 2025 Sep 10;11(37):eady5143. doi: 10.1126/sciadv.ady5143 (PMC12422195; doi:10.1126/sciadv.ady5143)
Supplement: Supplementary file 1 — Supplementary Text Figs. S1 to S21 Table S1 Legends for movies S1 to S15 [file sciadv.ady5143_sm.pdf]

Science Advances
AAAS

Supplementary Materials for

Rapid moving by liquid-amplified electrostatic rolling

Fei Jia *et al.*

Corresponding author: Jianglong Guo, guojianglong@hit.edu.cn; Jinsong Leng, lengjs@hit.edu.cn

Sci. Adv. **11**, eady5143 (2025)
DOI: 10.1126/sciadv.ady5143

The PDF file includes:

Supplementary Text
Figs. S1 to S21
Table S1
Legends for movies S1 to S15

Other Supplementary Material for this manuscript includes the following:

Movies S1 to S15

1 Supplementary Text

1.1 LAER driving mechanism theoretical considerations

In order to understand the LAER mechanism and its locomotion behavior, the force diagram of the large-scale rigid LAER roller is presented in Fig. S1. The forces on the LAER roller involve the gravity mg, the electrostatic pressures in the air side (P_{air} and $P_{\mathrm{air}}^{\mathrm{breakdown}}$) and the liquid side ($P_{\mathrm{oil}}$ and $P_{\mathrm{oil}}^{\mathrm{breakdown}}$), the support force N, the static friction f, the rolling resistance M_f, and the viscous resistance P_{r} produced by the viscosity effect of the dielectric liquid, which is proportional to the rolling speed, $P_{\mathrm{r}} \sim v$. It is assumed that the LAER roller is always in pure rolling.

Based on the Newton's second law of motion and the angular momentum theorem, we have the following governing equations (the dynamic equations for the LAER roller)

$$\mathbf{F}_{\mathrm{oil}} + \mathbf{F}_{\mathrm{air}} + \mathbf{F}_{\mathrm{I}} + \mathbf{F}_{\mathrm{r}} - m\mathbf{a} = \mathbf{0} \tag{S1}$$

$$M_{\mathrm{C}}(\mathbf{F}_{\mathrm{oil}}) + M_{\mathrm{C}}(\mathbf{F}_{\mathrm{air}}) + M_{\mathrm{C}}(\mathbf{F}_{\mathrm{I}}) + M_{\mathrm{C}}(\mathbf{F}_{\mathrm{r}}) - M_f - J\alpha = 0 \tag{S2}$$

where $\mathbf{F}_{\mathrm{oil}}$ and $\mathbf{F}_{\mathrm{air}}$ represent the electrostatic forces on the liquid side and air side, respectively; $\mathbf{F}_{\mathrm{I}}$ represents the resultant force of the support and the friction forces; $\mathbf{F}_{\mathrm{r}}$ denotes the viscous resistance resultant force; $\mathbf{a}$ and α is the acceleration and angular acceleration of the roller, respectively; $M_{\mathrm{C}}(\bullet)$ is the moment of forces ($\bullet$) about the center of gravity (C); $J = mR^2$ is the moment of inertia for the roller about the center of gravity, and R is the roller radius.

Eqs. S1 and S2 regulate the complete dynamics of LAER rollers, including the acceleration stage and the stable moving stage. Driven by the asymmetrically distributed electrostatic forces, the roller starts rolling from the rest and enters the acceleration stage; as the speed increases, the viscous resistance increases until the roller reaches an equilibrium state and switches into the stable moving stage.

1.1.1 Modeling the dynamic behavior of rolling from rest

To obatain the initial acceleration, the viscous resistance is zero due to $v = 0$. The rolling resistance is neglected and thus Eqs. S1 and S2 can be simplified as

$$\mathbf{F}_{\mathrm{oil}} + \mathbf{F}_{\mathrm{air}} + \mathbf{F}_{\mathrm{I}} - m\mathbf{a} = \mathbf{0} \tag{S3}$$

$$M_{\mathrm{C}}(\mathbf{F}_{\mathrm{oil}}) + M_{\mathrm{C}}(\mathbf{F}_{\mathrm{air}}) + M_{\mathrm{C}}(\mathbf{F}_{\mathrm{I}}) - J\alpha = 0 \tag{S4}$$

Because the direction of electrostatic pressure passes through the center of gravity, Eqs. S3 and S4 can be detailed as

$$F_{\mathrm{oil}}^{//} + F_{\mathrm{air}}^{//} - f - ma = 0 \tag{S5}$$

$$N - mg - F_{\mathrm{oil}}^{\perp} - F_{\mathrm{air}}^{\perp} = 0 \tag{S6}$$

$$fR - J\alpha = 0 \tag{S7}$$

where superscripts $//$ and $\perp$ represent the components of the resultant force in directions tangential and normal to the conductive surface, respectively. The relationship between a and α is $a = \alpha R$ for pure rolling. Then we have the acceleration of the LAER roller

$$a = \frac{F^{//}}{2m} = \frac{F_{\mathrm{oil}}^{//} - F_{\mathrm{air}}^{//}}{2m} \tag{S8}$$

where $F^{//}$ is the tangential component of the resultant electrostatic force or the net driving force.

The acceleration contributed by the net driving force allows the LAER roller to enter the initial acceleration stage from rest, which explains why the LAER roller enables rolling. In the initial acceleration stage, the net driving force is gradually balanced by the increasing viscous resistance with the speed. Then the acceleration gradually decreases to zero and the LAER roller enters the stable moving stage.

1.1.2 Modeling the dynamic behavior of the stable moving stage

To understand the locomotion behavior of LAER rollers in the stable moving stage and model the viscosity effect of dielectric liquid, we approximate the flow of the dielectric liquid propelled by LAER rollers using the Poiseuille flow (*35*, *36*) between two parallel plates. The relation between the average flow velocity (v_{l}) and the pressure drop (Δp) for the Poiseuille flow between two parallel plates is

$$v_{\mathrm{l}} = \frac{\Delta p H^2}{3\mu L} \tag{S9}$$

where H is half the distance between parallel plates, μ is the viscosity of the dielectric liquid, L is the length of the fluid-filled plates. Here H and L can be reasonably approximated as

$$H \approx \frac{z(\theta_{\mathrm{oil}}^{1})}{4} \approx \frac{(\theta_{\mathrm{oil}}^{1})^2 R}{4} \tag{S10}$$

and

$$L \approx \theta_{\text{oil}}^{1} R \tag{S11}$$

respectively, where θ_{oil}^{1} is the maximum angle of dielectric liquid distribution (see Figs. S1B and C), and $z = R(1 - \cos\theta)$ is the height of the liquid dielectric layer. In the stable moving stage, $a = 0$ and the net driving force is approximately balanced by the viscous resistance, so Δp can be represented as

$$\Delta p \approx \frac{2F^{//}}{z(\theta_{\text{oil}}^{1})W} \tag{S12}$$

where W is the width of the LAER roller (see Fig. 2A).

Substituting Eqs. S10−S12 into Eq. S9, the stable speed of the LAER roller, v (= v_1), can approximately be obtained as

$$v \approx \frac{F^{//}\theta_{\text{oil}}^{1}}{24\mu W} \tag{S13}$$

Eq. S13 can be used to better understand the effect of the investigated parameters on the speed of rigid LAER rollers (see Fig. 2) in the stable moving stage. However, due to the complexity of dielectric liquid dynamics under electric fields, the equation is only qualitatively analyzed.

1.2 Liquid-amplified electrostatic force

The liquid-amplified electrostatic force plays an important role in the LAER mechanism, providing the driving and adhesion force for the LAER rollers. Therefore, we performed theoretical derivation as well as experimental validations.

1.2.1 Theoretical derivation of the liquid-amplified electrostatic force

As shown in Figs. S1, we assume that all the dielectric liquid is retained on the liquid side and distributed from the contact point between the roller and the surface to θ_{oil}^{1} under the action of electric filed, and θ_{oil}^{1} can be obtained from

$$V_{\text{oil}} - R^2 W \int_0^{\theta_{\text{oil}}^{1}} (1 - \cos\theta)\cos\theta \mathrm{d}\theta = 0 \tag{S14}$$

where V_{oil} is the volume of dielectric liquid, R is the roller radius, W is the roller width, θ^1_{oil} is the maximum angle of dielectric liquid distribution, and θ is the independent variable. Here, we neglect the edge effect of the electric field and do not consider the effect of the ends of the roller. The electric potential difference of the fluid dielectric layer (oil or air) U_{m} and the insulating film U_{film} are

$$U_{\mathrm{m}} = U\frac{C_{\mathrm{film}}}{C_{\mathrm{m}} + C_{\mathrm{film}}} = U\frac{\epsilon_{\mathrm{film}} z}{t_{\mathrm{ring}}\epsilon_{\mathrm{m}} + z\epsilon_{\mathrm{film}}} \tag{S15}$$

$$U_{\mathrm{film}} = U - U_{\mathrm{m}} \tag{S16}$$

where the subscript m denotes the liquid or air side of the roller; U is the applied voltage; C_{m} and C_{film} denote the capacitance of the fluid dielectric layer and the insulating film, respectively; t_{film} denotes the thickness of the insulating film; $z = R(1-\cos\theta)$ is the height of the fluid dielectric layer, and ϵ_{m} and ϵ_{film} are the relative permittivity of the fluid dielectric and insulating film, respectively.

Based on the dielectric breakdown condition $U_{\mathrm{m}} = zE^{\mathrm{breakdown}}_{\mathrm{m}}$, the fluid dielectric breakdown region (0–θ^0_{m}) can be obtained as

$$\theta^0_{\mathrm{m}} = \arccos\left[(E^{\mathrm{breakdown}}_{\mathrm{m}} R\epsilon_{\mathrm{film}} - U_{\mathrm{m}}\epsilon_{\mathrm{film}} + E^{\mathrm{breakdown}}_{\mathrm{m}} t_{\mathrm{ring}}\epsilon_{\mathrm{m}})/(E^{\mathrm{breakdown}}_{\mathrm{m}} R\epsilon_{\mathrm{film}})\right] \tag{S17}$$

where $E^{\mathrm{breakdown}}_{\mathrm{m}}$ is the breakdown strength of the fluid dielectric. According to Eq. S17, θ^0_{air} and θ^0_{oil} as a function of U are shown in Fig. S2A, and θ^1_{oil} ($= \theta^1_{\mathrm{air}}$) is the maximum angle of the dielectric liquid distribution, and is obtained from the geometric relationship in Eq. S14. It can be observed that electric breakdown occurs early in the air side and the breakdown range θ^0_{air} increases rapidly with increasing U, which is attributed to the fact that dielectric breakdown strength of the air (3 kV/mm), $E^{\mathrm{breakdown}}_{\mathrm{air}}$, is around 15% of the dielectric liquid (20 kV/mm) (*32*). When $U = 10.00$ kV, the entire region $0 - \theta^1_{\mathrm{air}}$ in the air side is in the case of dielectric breakdown.

The electric field strength, E_{m}, in the fluid dielectric (oil or air) increases with increasing U until electric breakdown occurs and it is assumed that the maximum field strength, $E^{\mathrm{breakdown}}_{\mathrm{m}}$, can be maintained after the dielectric breakdown (*32*). The electrostatic pressures in the dielectric breakdown ($0 - \theta^0_{\mathrm{m}}$) and non-breakdown ($\theta^0_{\mathrm{m}} - \theta^1_{\mathrm{m}}$) regions are $P^{\mathrm{breakdown}}_{\mathrm{m}}$ and P_{m}, respectively, and can be expressed as

$$P^{\mathrm{breakdown}}_{\mathrm{m}} = \frac{1}{2}\epsilon_{\mathrm{m}}\epsilon_0(E^{\mathrm{breakdown}}_{\mathrm{m}})^2 \tag{S18}$$

$$P_{\mathrm{m}} = \frac{1}{2}\epsilon_{\mathrm{m}}\epsilon_0(E_{\mathrm{m}})^2 \tag{S19}$$

where ϵ_0 is the vacuum permittivity.

The tangential $F_{\mathrm{m}}^{//}$ and normal $F_{\mathrm{m}}^{\perp}$ components of the electrostatic force in the fluid dielectric can be expressed as

$$F_{\mathrm{m}}^{//} = RW\left(\int_0^{\theta_{\mathrm{m}}^0} P_{\mathrm{m}}^{\mathrm{breakdown}}\sin\theta\mathrm{d}\theta + \int_{\theta_{\mathrm{m}}^0}^{\theta_{\mathrm{m}}^1} P_{\mathrm{m}}\sin\theta\mathrm{d}\theta\right) \tag{S20}$$

$$F_{\mathrm{m}}^{\perp} = RW\left(\int_0^{\theta_{\mathrm{m}}^0} P_{\mathrm{m}}^{\mathrm{breakdown}}\cos\theta\mathrm{d}\theta + \int_{\theta_{\mathrm{m}}^0}^{\theta_{\mathrm{m}}^1} P_{\mathrm{m}}\cos\theta\mathrm{d}\theta\right) \tag{S21}$$

Then $F^{//}$ can be expressed as:

$$\begin{aligned} F^{//} = F_{\mathrm{oil}}^{//} - F_{\mathrm{air}}^{//} &= RW\left(\int_0^{\theta_{\mathrm{oil}}^0} P_{\mathrm{oil}}^{\mathrm{breakdown}}\sin\theta\mathrm{d}\theta + \int_{\theta_{\mathrm{oil}}^0}^{\theta_{\mathrm{oil}}^1} P_{\mathrm{oil}}\sin\theta\mathrm{d}\theta\right) \\ &- RW\left(\int_0^{\theta_{\mathrm{air}}^0} P_{\mathrm{air}}^{\mathrm{breakdown}}\sin\theta\mathrm{d}\theta + \int_{\theta_{\mathrm{air}}^0}^{\theta_{\mathrm{air}}^1} P_{\mathrm{air}}\sin\theta\mathrm{d}\theta\right) \end{aligned} \tag{S22}$$

The electrostatic force in the air side, F_{air}, is limited to a very small value, as shown in Fig. S2B. The high breakdown strength and large permittivity of the dielectric liquid, as two complementary factors, jointly amplify the electrostatic force in the liquid side (F_{oil}).

1.2.2 Experimental validation for the theoretical electrostatic force

Two customized experimental setups were constructed to characterize the tangential and normal electrostatic forces (see the insets of Figs. S3A and S4A, respectively). These quasi-static characterization experiments were unable to investigate the effect of liquid viscosity. In these characterization tests, the default settings were D = 45.0 mm, t_{ring} = 2.0 mm, W = 75.0 mm, U = 9.00 kV, μ = 200 mPa·s and V_{oil} = 1.00 ml. The roller surface texture is the 'medium' texture. Each test was repeated three times.

Here, two liquid-amplified electrostatic models were considered to compare with the experimental results, one without considering the dielectric breakdown of the liquid, and the other considering the dielectric breakdown of the liquid and adopting the assumptions about the dielectric breakdown of the liquid. The electric field strength, E_{oil}, in the liquid increases with increasing U until dielectric breakdown occurs and it is assumed that the maximum field strength, $E_{\mathrm{oil}}^{\mathrm{breakdown}}$, can be

maintained after the dielectric breakdown (*32*). The relative permittivity and breakdown strength of the used silicone oil were determined to ~2.7 and ~20 kV/mm (*32*) (matching the silicone oil in most experiments), respectively. The relative permittivity and breakdown strength of the air are 1 and 3 kV/mm, respectively. The thickness and the relative permittivity of the PVC tape used were experimentally obtained as ~0.18 mm and ~7.1 at 100 Hz. Its breakdown strength was given by the manufacturer as ~44 kV/mm. In conclusion, the experimental results are in reasonable agreement with the model considering liquid breakdown as shown in Figs. S3 and S4.

As shown in the inset of Fig. S3A, a load cell was used to measure the net driving force, $F^{//}$, which equals to F_S, the force exerted by the load cell to the roller. This is because that the roller was restrained by the load cell and was in static equilibrium. The force diagram of the roller is depicted in inset of Fig. S3A, and the equilibrium equations of the roller in static equilibrium can be obtained as

$$F^{//}_{\text{oil}} - F^{//}_{\text{air}} - f - F_S = 0 \tag{S23}$$

$$M_C(f) = 0 \tag{S24}$$

Then $F_S = F^{//}$ is readily reached.

To ensure that the entire dielectric liquid was fully utilized, the experimental procedure was to first apply an electric field and then add the liquid to the liquid side. Under dielectrophoretic force, the whole liquid was attracted to the liquid side. A typical $F^{//}$−time curves of the large-scale rigid LAER roller is demonstrated in Fig. S3A.

At the initial moment, there was a rapid increase followed by a slow increase in $F^{//}$. The rapid increase was due to the rapid addition of dielectric liquid on the liquid side. The slow increase maybe due to the gradual expansion of the liquid in the direction of roller width and height under the action of electric field. Then $F^{//}$ gradually decreased until the liquid was symmetrically distributed. This was because that the dielectric liquid flowed from the liquid side via the roller ends to the air side. This phenomenon should not occur in the case of stable and continuous rolling of LAER rollers, because the rolling behavior prevents the liquid from entering the air side. Therefore, the maximum $F^{//}$ were selected for comparison with theoretical results, as illustrated in Figs. S3B−D.

Fig. S3B depicts the effect of voltage on the tangential electrostatic force, which increased with increasing voltage. Fig. S3C illustrates the effect of liquid volume on the tangential electrostatic force, which first increased and then remained almost constant with increasing liquid volume. Fig.

S3D shows the effect of roller diameter on the tangential electrostatic force, and the effect was almost negligible.

In the inset of Fig. S4A, the LAER roller with a force cell was mounted on a linear stage. At the beginning, a preload force of roughly 4 N was applied to the roller to ensure complete contact with the base. Then, a voltage was applied, and twice the studied volume of liquid was added to both sides of the roller, and the roller was lifted from the base at a speed of 10 μ/s by the linear stage. The normal electrostatic force-time curve (see Fig. S4A) was obtained and the maximum normal electrostatic force was selected. The normal electrostatic force in the absence of liquid was then measured. Finally, the required normal electrostatic force was the average of the above two.

Fig. S4B depicts the effect of voltage on the normal electrostatic force, which increased with increasing voltage. Fig. S4C illustrates the effect of liquid volume on the normal electrostatic force, which first increased and then remained almost constant with increasing liquid volume. Fig. S4D shows the effect of roller diameter on the normal electrostatic force, which increased with increasing roller diameter. From Figs. S3 and S4, it can be seen that the normal electrostatic force was significantly greater than the tangential electrostatic force.

1.3 Scaling analysis of rigid LAER rollers

A scaling analysis was performed to obtain the parameter group which governs the locomotion behavior of rigid LAER rollers. In the volume-dominated regime, a small volume of dielectric liquid is used so that the entire liquid is in dielectric breakdown under the high applied voltage. From Eq. S14, we can obtain

$$\theta_{\text{oil}}^{1} \propto \left(\frac{V_{\text{oil}}}{R^2 W}\right)^{\frac{1}{3}} \tag{S25}$$

where θ_{oil}^{1} is the maximum angle of dielectric liquid distribution and R is the roller radius. In Eq. S22, the contribution to $F^{//}$ from the air side is small and can be neglected (see Fig. S2B), so we can approximately obtain

$$F^{//} \propto RW\epsilon_{\text{oil}}^{*}(E_{\text{oil}}^{\text{breakdown}})^2(\theta_{\text{oil}}^{1})^2 \tag{S26}$$

where $\epsilon_{\text{oil}}^{*}$ denotes the permittivity of liquid dielectric and $E_{\text{oil}}^{\text{breakdown}}$ denotes the breakdown strength of dielectric liquid. Substituting Eqs. S25 and S26 into Eq. S13, the governing parameter group about the crawling speed in the volume-dominated regime can be determined as

$$\frac{v}{E_{\text{oil}}^{\text{breakdown}}(\epsilon_{\text{oil}}^{*})^{\frac{1}{2}}/\rho^{\frac{1}{2}}} \propto \frac{E_{\text{oil}}^{\text{breakdown}}(\epsilon_{\text{oil}}^{*})^{\frac{1}{2}}\rho^{\frac{1}{2}}}{W}\frac{V_{\text{oil}}}{\mu R} \tag{S27}$$

which implies that the speed is proportional to the dielectric liquid volume, and inversely proportional to liquid viscosity and roller radius.

In the voltage-dominated regime, the used liquid volume $(0-\theta_{\text{oil}}^{1})$ is larger than the maximum liquid volume that can be retained on the liquid side by the electric field. The excess liquid can not be retained on the liquid side, which results in the fact that the increase of liquid volume has almost no effect on the speed. A portion $(0-\theta_{\text{oil}}^{0})$ of the liquid is in breakdown (see Fig. S1B). Here, only the dominant term (first term) in Eq. S22 is considered, and we can approximately obtain

$$F^{//} \propto RW\epsilon_{\text{oil}}^{*}(E_{\text{oil}}^{\text{breakdown}})^{2}(\theta_{\text{oil}}^{0})^{2} \tag{S28}$$

Considering the dielectric breakdown condition, θ_{oil}^{0} can be derived as

$$(\theta_{\text{oil}}^{0})^{2} \propto \frac{U}{E_{\text{oil}}^{\text{breakdown}}R} \tag{S29}$$

Substituting Eq. S29 into Eq. S28 can obtain

$$F^{//} \propto W\epsilon_{\text{oil}}^{*}E_{\text{oil}}^{\text{breakdown}}U \tag{S30}$$

which suggests that the net driving force is proportional to the voltage.

According to electrowetting theory (*37*) and considering the equilibrium of surface tension and gravity for the dielectric liquid, the effective volume of dielectric liquid V_{oil}^{*}, can be determined as

$$V_{\text{oil}}^{*} \propto \frac{C_{\text{H}}WU^{2}}{\rho g} \tag{S31}$$

where C_{H} is a constant related to electro-capillarity (*37*), ρ is the density of dielectric liquid and is constant, and g is the gravitational acceleration. Combining with Eqs. S14 and S31, θ_{oil}^{1} can be obtained as

$$\theta_{\text{oil}}^{1} \propto \left(\frac{C_{\text{H}}U^{2}}{\rho g R^{2}}\right)^{\frac{1}{3}} \tag{S32}$$

Substituting Eqs. S30 and S32 into Eq. S13, the governing parameter group about crawling speed in the voltage-dominated regime can be determined as

$$\frac{v}{E_{\mathrm{oil}}^{\mathrm{breakdown}}(\epsilon_{\mathrm{oil}}^{*})^{\frac{1}{2}}/\rho^{\frac{1}{2}}} \propto \frac{(C_{\mathrm{H}})^{\frac{1}{3}}\rho^{\frac{1}{6}}(\epsilon_{\mathrm{oil}}^{*})^{\frac{1}{2}}}{g^{\frac{1}{3}}}\frac{U^{\frac{5}{3}}}{\mu R^{\frac{2}{3}}} \tag{S33}$$

which reveals that the speed is independent of the dielectric liquid volume and proportional to $U^{\frac{5}{3}}$ when the dielectric liquid volume is large. $E_{\mathrm{oil}}^{\mathrm{breakdown}}(\epsilon_{\mathrm{oil}}^{*})^{\frac{1}{2}}/\rho^{\frac{1}{2}}$ represents the speed limit, depending on the dielectric properties of the dielectric liquid, at which the voltage is high enough to make the entire dielectric droplet in the dielectric breakdown. The scaling relation of Eq. S33 in the voltage-dominated regime, was consistent with the experimental results, as demonstrated in Fig. 2H.

1.4 Versatile moving of LAER rollers

Versatile moving capabilities of rigid LAER rollers are shown in Fig. S5 and Figs. 4A and B. Fig. S5A shows the forward moving, the basic locomotive mode, of LAER rollers (see Movie S1). Fig. S5B demonstrates that a LAER roller with two different surface textures along the axial direction can be used to achieve controlled turning (see Movie S2), because the relatively rough roller surface can provide a greater friction than the smooth roller surface, thus creating a horizontal torque to achieve turning towards the rough side. Circular turning can also be achieved by using a tapered LAER structure (see Movie S2), as shown in Fig. 4B. Apart from forward moving, turning, circular moving, the centimeter-scale rigid LAER roller can further be used for circular climbing, as shown in Fig. 4A (see Movie S3). Fig. 1C presents the prototypes of centimeter-scale and submillimeter-scale rigid LAER rollers. The circular climbing was also realized by submillimeter-scale rigid LAER rollers as shown in Fig. S5C (see Movie S3).

Versatile moving capabilities of flexible LAER rollers are shown in Fig. S6. Figs. S6A and B demonstrates climbing on 60° slopes and vertical walls, respectively, of the centimeter-scale flexible LAER rollers (see Movie S4). Fig. 4C demonstrates forward moving of a large-scale LAER roller by directly using an unopened readily available Coca-Cola can (330 ml) at a speed of 0.12 BL/s under U = 4.75 kV, μ = 20 mPa·s and V_{oil} = 0.60 ml (see Movie S7). Fig. S6C demonstrates forward moving of a centimeter-scale flexible LAER roller carrying a weight of ~1278.600 g (load-to-weight ratio of ~121) at a speed of 0.07 BL/s under U = 7.00 kV, μ = 50 mPa·s, V_{oil} = 1.30 ml, D = 66.2 mm and W = 167.8 mm (see Movie S7). The maximum load to weight ratio ~121 was realized by filling a can with liquid metal. Fig. 4D demonstrates the vertical climbing of a centimeter-scale flexible LAER roller carrying a payload of ~59.630 g (load-to-weight ratio

of ~16) at a speed of 0.60 BL/s under U = 10.00 kV, μ = 20 mPa·s, V_{oil} = 1.50 ml and D =53.0 mm (see Movie S7). The maximum load to weight ratio ~16 was realized by installing plasticine embedded 5 mm diameter steel balls inside the LAER flexible roller. Both of these loading methods caused the flexible rollers to become stiffer.

Fig. S6D demonstrates circular climbing (see Movie S3) of the centimeter-scale flexible LAER roller under U = 6.00 kV, μ = 20 mPa·s, V_{oil} = 1.50 ml and D = 53.0 mm. Fig. S6E demonstrates plane-to-plane transitioning (see Movie S4) of the centimeter-scale flexible LAER roller under U = 4.60 kV, μ = 20 mPa·s and D = 66.2 mm. The plane-to-plane transitioning of flexible rollers was realized by the following method. When a flexible roller moving on floors encountered a wall, the potential difference between the conductive floor and the roller was removed, and the potential difference between the conductive wall and the roller was applied. Then the dielectric liquid was added to the slit between the roller and the wall, and the roller achieved plane-to-plane transitioning from the floor to the wall.

1.5 Displacements and speeds of LAER rollers

1.5.1 Displacements of large-scale rigid LAER rollers

The displacement-time curves corresponding to the speed characterization results in Fig. 2 are presented in Fig. S7. Here, the default setting were U = 9.00 kV, V_{oil} = 1.00 ml, μ = 200 mPa·s, D = 45.0 mm and t_{ring} = 2.0 mm. A laser displacement sensor was used to record the displacement of rollers (see the inset of Fig. S7A). Displacement-time curves for different applied voltages are shown in Fig. S7A. The displacement had a roughly uniform increase over time when the voltage was 5−15 kV. The roller moved a certain distance and then stopped moving, due to the disappearance of asymmetric liquid distribution, when the voltage was below the voltage threshold (5.00 kV). Displacement-time curves for different dielectric liquid volumes and viscosities are depicted in Figs. S7B and C, respectively. All displacement-time curves over the threshold had a stable linear segment. 0.1 ml of dielectric liquid failed to move the roller.

1.5.2 Displacements of submillimeter-scale rigid LAER rollers

The displacement-time curves of submillimeter-scale rigid rollers during crawling are shown in Figs. S8A−C. The displacement-time curves for different voltages in 5−10 kV are shown in Fig. S8A. The displacement-time curves for different liquid volume in 10−300 μl are shown in Fig.

S8B. The displacement-time curves for different liquid viscosities in 50−500 mPa·s are shown in Fig. S8C. Three repeated displacement-time curves for the fastest crawling, vertical and inverted climbing speeds observed in Fig. S8A−C, are depicted in Figs. S8D−F, respectively. It can be seen that there were an acceleration stage and a stable moving stage and that the trajectory was straight. All displacement-time curves demonstrated in Fig. S8 had a stable linear segment.

1.5.3 Displacements and speeds of large-scale flexible LAER rollers

Here, the default setting were U = 6.00 kV, V_{oil} = 0.15 ml, μ = 20 mPa·s and D = 53.0 mm. The displacement-time curves of large-scale flexible rollers for different applied voltages are shown in Fig. S9A. The displacement of the flexible roller increased uniformly with time at an applied voltage of 4-10 kV. The displacement-time curves of large-scale flexible rollers for different dielectric liquid volumes and viscosities are illustrated in Figs. S9B and C, respectively. All displacement-time curves demonstrated in Fig. S9 had a stable linear segment.

1.6 Slow-motion (0.05×) playback of the rolling process of the submillimeter-scale rigid roller

A partial blackening treatment was applied to one end of the submillimeter-scale roller, as illustrated in Fig. S10A. The purpose was to observe whether the roller was rolling or translating under the action of an electric field. A high-speed camera (Revealer X113M, CHINA) was used to record the moving behavior of the submillimeter-scale roller. The capture frame rate was 500 fps. Fig. S10B shows the snapshots of the roller moving, where U = 8.00 kV, V_{oil} = 10 µl and μ = 10 mPa·s. From left to right are frames 1, 31, and 63. It can be seen that the black marker was not visible in frames 1 and 63. In frame 31, the black marker was visible. This indicates that the submillimeter-scale roller was rolling rather than translating under the action of an electric field. Movie S6 presents this slow-motion (0.05×) playback of the rolling process of the submillimeter-scale LAER roller. The playback frame rate is 25 fps. The experimental condition was U = 8.00 kV, V_{oil} = 10 µl and μ = 10 mPa·s.

1.7 Effect of residual liquid and pre-placed position of dielectric liquid

In the speed characterization experiments for LAER rollers, the liquid dielectric on the surfaces of the rollers and the encapsulated conductive surface were wiped off with non-woven fabrics after

each trial, to ensure standardization and reproducibility of the experiments. The crawling speeds of the submillimeter-scale and large-scale LAER rollers were tested with U = 6.00 kV, μ = 200 mPa·s and V_{oil} = 10 μl and with U = 9.00 kV, μ = 200 mPa·s, V_{oil} = 1.00 ml and D = 45.0 mm, respectively, under the condition of liquid dielectric retained on the roller surfaces. As shown in Fig. S11, the speed variation of sub-millimeter rollers caused by residual liquid was less than 16.5%. The speed variation of large-scale rollers caused by residual liquid was less than 3.8%. The sub-millimetre rollers were easily affected by experimental errors due to their small size. It can be seen that the liquid dielectric remained on the roller surfaces had relatively minor effect on the crawling speed of the rigid LAER rollers.

The effect of the liquid pre-placed position on the crawling speed of rigid LAER rollers was experimentally investigated. Under U = 6.00 kV, μ = 200 mPa·s and V_{oil} = 10 μl, we experimentally examined the effect of the liquid pre-placed position at left, middle, and right on the crawling speed of submillimeter-scale LAER rollers, and the results are shown in Fig. S12A. It can be seen that the speed variation of sub-millimeter rollers caused by liquid pre-placed position was less than 7.5%. This maybe attributed to the fact that the liquid was expanded rapidly in the roller width direction on the liquid side when an electric field was applied. Fig. S12B depicts the effect of the liquid pre-placed position on the crawling speed of the large-scale rigid LAER rollers at U = 9.00 kV, μ = 200 mPa·s, V_{oil} = 1.00 ml and D = 45.0 mm. It can be seen that the speed variation of large-scale rollers caused by liquid pre-placed position was less than 11.2%. The crawling speed with the middle pre-placed position was slightly faster than the left and right, and the left and right pre-placed positions had about the same crawling speeds.

1.8 Locomotive efficiency of LAER rollers

We measured the operating current of LAER rollers and obtained their power consumption for different applied voltages. Fig. S13 illustrates the current as a function of time of large-scale rigid LAER rollers for the same travel distance 500 mm. Figs. 2F and 3H illustrate the average current and power consumption of the large-scale and small-scale rigid LAER rollers, respectively, during crawling for different voltages. Fig. S14 shows the average current and power consumption of the large-scale flexible LAER rollers during crawling for different voltages. It can be seen that the average current and power consumption of these LAER rollers increased as the applied voltage increased, and the forward moving speed of these LAER rollers also increased with increasing

applied voltage as shown in Figs. 2C and 3C.

We compared the locomotive efficiency of these LAER rollers at different voltages by calculating the cost of transport (CoT):

$$\mathrm{CoT} = \frac{P_{\mathrm{avg}}}{mgv_{\mathrm{avg}}} \tag{S34}$$

where P_{avg} is the average power consumption of LAER rollers, v_{avg} is the stable speed of LAER rollers, m is roller mass, and g is the acceleration due to gravity. The CoTs as a function of voltage for large-scale rigid and flexible rollers and submillimeter-scale rigid rollers are plotted in Fig. S15. For large-scale rigid LAER rollers, CoT increased with increasing applied voltage. For large-scale flexible LAER rollers, CoT was almost unaffected by the applied voltage. For submillimeter-scale rigid LAER rollers, CoT increased with increasing applied voltage.

1.9 Serial LAER robot with liquid regulating modules

Fig. 4E and Movie S8 shows the reciprocating moving and short stops of the serial LAER robot with liquid regulating modules. The isometric view of the serial LAER robot with liquid regulating modules is shown in Fig. S16A. Note that here the two rollers were electrically isolated. First, the voltage was only applied to the right roller, and some dielectric liquid was added to the left side of the right LAER roller. The serial LAER robot moved to the left. Then, we removed the voltage from the right roller, and the serial LAER robot quickly stopped moving and paused for 9.58 s. The voltage was applied again, and the serial LAER robot immediately continued to move to the left. Conversely, when the voltage and dielectric liquid were only applied to the left roller, the robot moved to the right, as shown in Movie S8. Fig. S16B shows the displacement-time curve of reciprocating moving and short stops of the serial LAER robot. There were three short stops here, which were 9.58 s, 20.56 s, and 8.01 s, from left to right.

The two LAER rollers were connected in series via a 3D-printed frame, but the two LAER rollers were electrically isolated. This means that different voltages can be applied to the two rollers. The outlet ports (needles) of two independent liquid regulating modules were installed between rollers, as shown in the Fig. S16A. The two LAER rollers were stainless steel cylinders with a diameter of 12 mm, a wall thickness of 0.5 mm, and a width of 50 mm. The diameter of the carbon rod used was 2 mm. In the experiment of Fig. 4E and Movie S8, a voltage of 5 kV and a dielectric liquid viscosity of 350 mPa·s were used.

1.10 Parallel LAER robot with liquid regulating modules

As shown in Fig. 4F and Movie S9, initially, the voltage and the identical dielectric liquid volumes were applied to both rollers. The parallel LAER robot moved forward. Then, the outer roller had a significantly greater dielectric liquid volume than the inner roller, which was applied through the liquid regulating modules. Accordingly, the parallel LAER robot turned. Finally, the dielectric liquid volume of the inner roller gradually increased. The moving direction of the parallel LAER robot was adjusted to be opposite to the initial direction.

Figs. S17A and B show the prototype and the isometric view of the parallel LAER robot with liquid regulating modules, respectively. The parallel LAER robot mainly consisted of two LAER rollers and two liquid regulating modules. Two LAER rollers were connected in parallel via a carbon rod, and each roller could rotate freely around the rod. The carbon rod and the rollers were electrically connected. This implies that the identical voltage was applied to the rollers. Two independent liquid regulating modules were fixed ahead of the rollers through a white 3D-printed frame. This allows independent adjustment of the dielectric liquid volume for each LAER roller. The differential dielectric liquid volumes between the two rollers resulted in distinct rolling speeds. This speed difference caused the whole structure to turn towards the slower side in a controlled manner. Four small wheels were installed below the 3D-printed frame to keep it level at all times. Four small wheels were miniature bearings with an outer diameter of 3 mm, an inner diameter of 1 mm, and a height of 1 mm.

The two LAER rollers were stainless steel cylinders with a diameter of 10 mm, a wall thickness of 0.5 mm, and a width of 40 mm. The diameter of the carbon rod used was 2 mm. Both LAER rollers and the 3D-printed frame can rotate freely around the carbon rod. The needles should be as close as possible to the rollers and base. As shown in the Fig. S17C, the distance between the needles and the rollers (base) was 0.4 mm in the design. The top view of the parallel LAER robot is shown in Fig. S17D. In Fig. 4F and Movie S9, the applied voltage was 7 kV, and the viscosity of the dielectric liquid was 350 mPa·s.

1.11 Single-wheeled untethered LAER robot

A single-wheeled untethered LAER robot was designed and developed. Fig. S18A shows the detailed design and fabrication process of the single-wheeled untethered LAER robot. The single-wheeled untethered LAER robot mainly consisted of a cross-shaped voltage-boosting device and

three conductive tubes. The voltage-boosting device included a Li-Po battery, wireless switch, a voltage booster module, high voltage converter (listed in Table S1) and three printed circuit boards (PCBs). The shapes of the three PCBs are shown in Fig. S18A (a). The hollowed-out parts in the center of the PCBs were for installing the above electronic modules. All electronic modules were installed in the center of the cross-shaped printed circuit board. Some pads were provided on the three PCBs for soldering the three PCBs together to form a cross-shaped PCB, as illustrated in Fig. S18A (b). High-voltage (HV) positive and negative outputs were located at the edges of the cross-shaped PCB. The voltage-boosting device can boost the battery's 3.7 V voltage to an output voltage of 6.00 kV.

All three conductive tubes had an outer diameter of 25 mm and a wall thickness of 0.5 mm. The conductive tube in the middle was an 80 mm wide carbon tube. Both ends were 5 mm wide stainless steel tubes. Three conductive tubes were mounted on the cross-shaped PCB, as shown in Fig. S18A (c). The carbon tube was connected to the positive output, and the two stainless steel tubes were connected to the negative output. The space between the tubes was 10 mm. A dielectric layer (two layers of Polyvinyl Chloride tapes) was used to encapsulate the surface of the carbon tube, as shown in Fig. S18A (d). However, the stainless steel tubes could always be in contact with the conductive surface. Therefore, a potential difference was created between the carbon tube and the conductive surface.

Fig. 5A and Movie S10 show the single-wheeled untethered LAER robot moving on stainless steel surfaces. The used viscosity and volume of the dielectric liquid were 10 mPa·s and 1.00 ml, respectively. Fig. S18B illustrate the displacement as a function time for the single-wheeled untethered LAER robot. The average speed was 165.5 mm/s (6.62 BL/s). Fig. S18C shows the speed as a function of time for the single-wheeled untethered LAER robot. It can be seen that the maximum instantaneous speed was 286.90 mm/s (11.48 BL/s). The displacement and speed of the single-wheeled untethered LAER robot were obtained by software "Tracker".

1.12 Two-wheeled untethered LAER robot

Fig. S19 illustrates the electronic design of the two-wheeled untethered LAER robot for environmental monitoring and detection. The electronic design included a 3.7 V Li-Po battery, a wireless switch, a voltage booster, a high voltage converter and a camera, as listed in Table S1. This electronic design could boost an voltage input of 3.7 V to a high-voltage output of 6.00 kV. The frame of the

two-wheeled untethered LAER robot was made of a printed circuit board (PCB). This PCB enabled electrical connection between the high voltage converter and the carbon rod. A small conductive round hole was opened on the PCB to accommodate the carbon rod. The small conductive hole was in sliding contact with the carbon rod. The flexible roller, cylindrical stiff foams and the carbon rod were bonded together as a single unit. The electrical connection between the carbon rod and the flexible roller was readily achievable.

1.13 LAER-based linear actuator

The LAER structure consists of two electrodes, rollers and conductive plates. One method is that the conductive plate is fixed, and the roller moves under an electric field. Another method is that the roller is hinged but can rotate, and the conductive plate moves under an electric field. Therefore, we designed and developed a LAER-based linear actuator, as shown in Fig. 5C and Movie S11. Fig. S20 describes the structural design of the LAER-based linear actuator.

Two LAER rollers were hinged on the base in series, and can rotate freely. An insulated conductive plate, as the linear moving plate, was placed on the rollers. Apply a voltage of 6 kV and some dielectric liquid to the right (left) LAER roller, and the linear moving plate moved to the right (left). Movie S11 shows the reciprocating motion of the LAER-based linear actuator with a stroke length of 22.62 mm. It is convenient to increase the stroke length by increasing the number of serial rollers and the length of the linear moving plate.

The two LAER rollers were stainless steel cylinders with a diameter of 12 mm, a wall thickness of 0.5 mm, and a width of 50 mm. The distance between the two rollers was 120 mm. The linear moving plate placed on the two rollers was an insulated conductive plate with length 220 mm and width 80 mm. Two independent liquid regulating modules can adjust the dielectric liquid volume for two rollers. In the experiment of Fig. 5C and Movie S11, a voltage of 6.00 kV and a dielectric liquid viscosity of 350 mPa·s were used.

1.14 Environmental robustness of LAER rollers

We prepared stainless steel plates, rusty iron plates, and stainless steel plates with water droplets or obstacles. Under the same conditions, the locomotive ability of the same LAER roller on these surfaces was qualitative observed, as shown in Fig. S21 and Movie S15. The LAER roller was a carbon tube with an outer diameter of 25 mm, a wall thickness of 0.5 mm, and a width of 80 mm.

The outer surface of the carbon tube was insulated by two layers of PVC tapes. The viscosity and volume of the dielectric liquid were 350 mPa·s and 1.00 ml, respectively. The applied voltage was 6.50 kV.

The moving of the LAER roller on the stainless steel plate, as a reference case, is shown in Fig. S21A. The LAER roller successfully moved from the left side (Fig. S21A (a)) to the right side (Fig. S21A (b)). Fig. S21B illustrates the rolling of the LAER roller on the rusty iron plates. When the roller reached the position shown in Fig. S21B (b), it stopped rolling, may be due to too much rust powders adsorbed onto the roller surface under the action of the electric field. In Fig. S21C (a), we placed four 20 μl dyed water droplets at intervals on the stainless steel plate. When the roller moved to position shown in Fig. S21C (b), a clear decrease in its rolling performance could be observed.

In Fig. S21D (a), we placed three small conductive obstacles at intervals on the stainless steel plate. The obstacles were 10 mm long and wide. Their heights from left to right were 0.1, 0.2, and 0.3 mm, respectively. When faced with the 0.1 mm high obstacle, the roller briefly got stuck, then crossed the obstacle. The rolling performance was weakened. Finally, it was unable to cross the 0.2 mm obstacle in Fig. S21D (b). In conclusion, water droplets, rust, and small obstacles on conductive surfaces would weaken the actuation and moving performance of the LAER rollers.

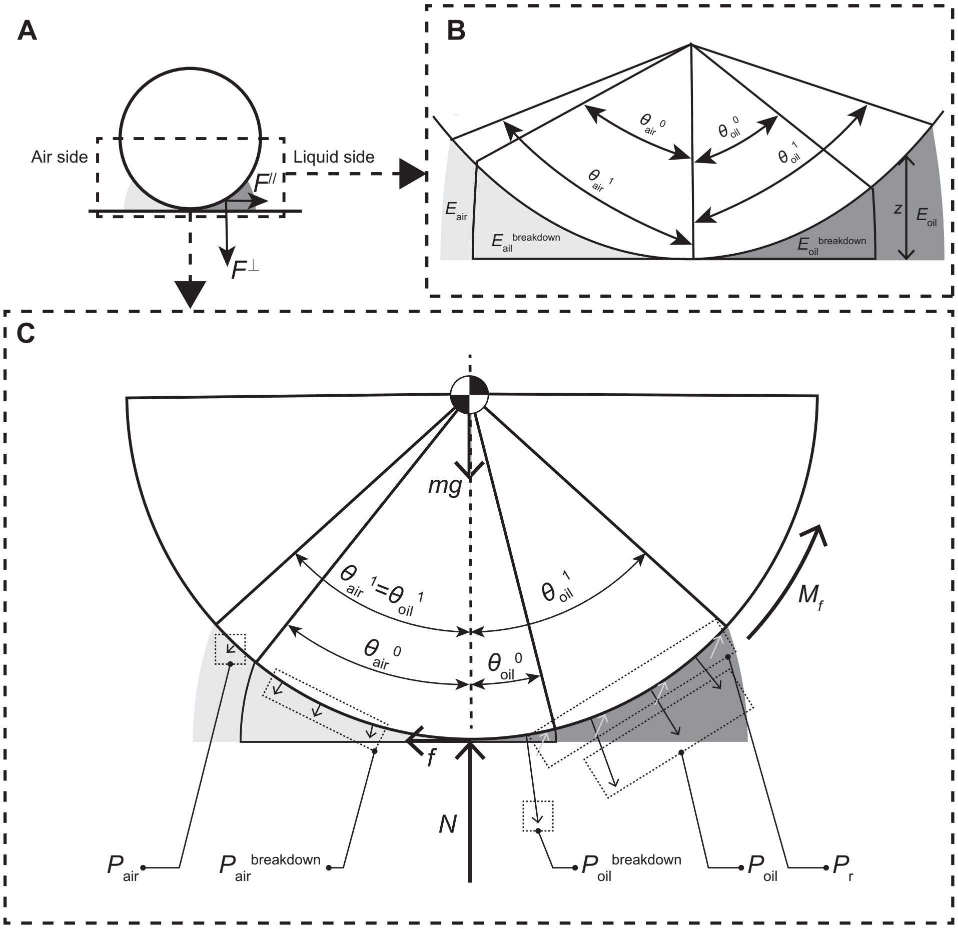

Figure S1: **Electric field and force analysis of the rigid LAER roller. A** Schematic diagram of the rigid LAER structure. **B** Distribution of electric field strength in the fluid (dielectric liquid and air). **C** Force diagram of the rigid LAER roller.

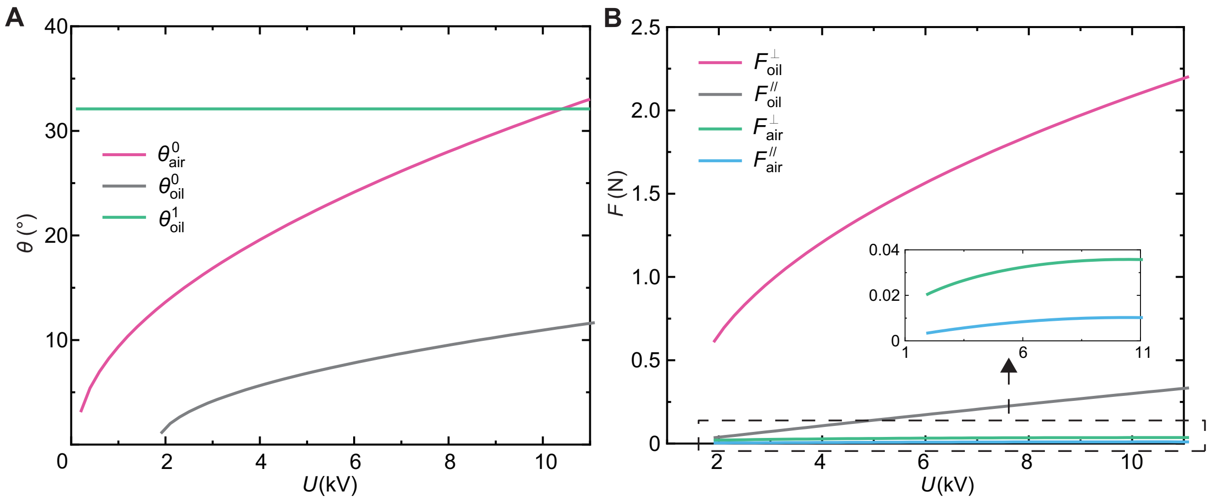

Figure S2: **Theoretical results for liquid and air sides. A** Theoretical relationship between breakdown region and applied voltage. **B** Theoretical relationship between electrostatic forces and applied voltage.

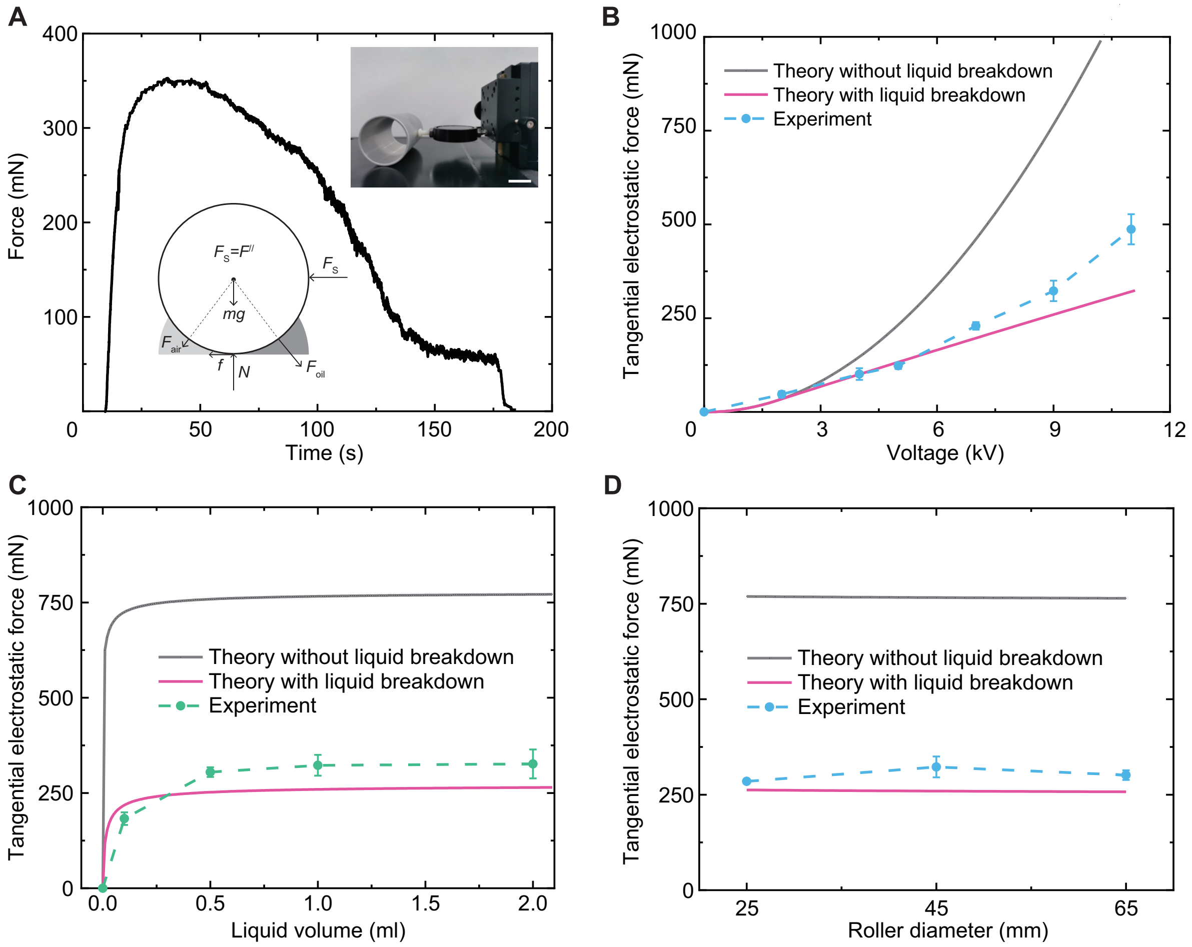

Figure S3: **Experimental and theoretical results on the tangential electrostatic force of centimeter-scale rigid LAER rollers. A** Tangential electrostatic force versus time obtained from experiments. The insets are the related experimental setup and force diagram of the roller in the experimental setup. **B** Effect of voltage on tangential electrostatic force. **C** Effect of liquid volume on tangential electrostatic force. **D** Effect of roller diameter on tangential electrostatic force. Scale bar denotes 20 mm.

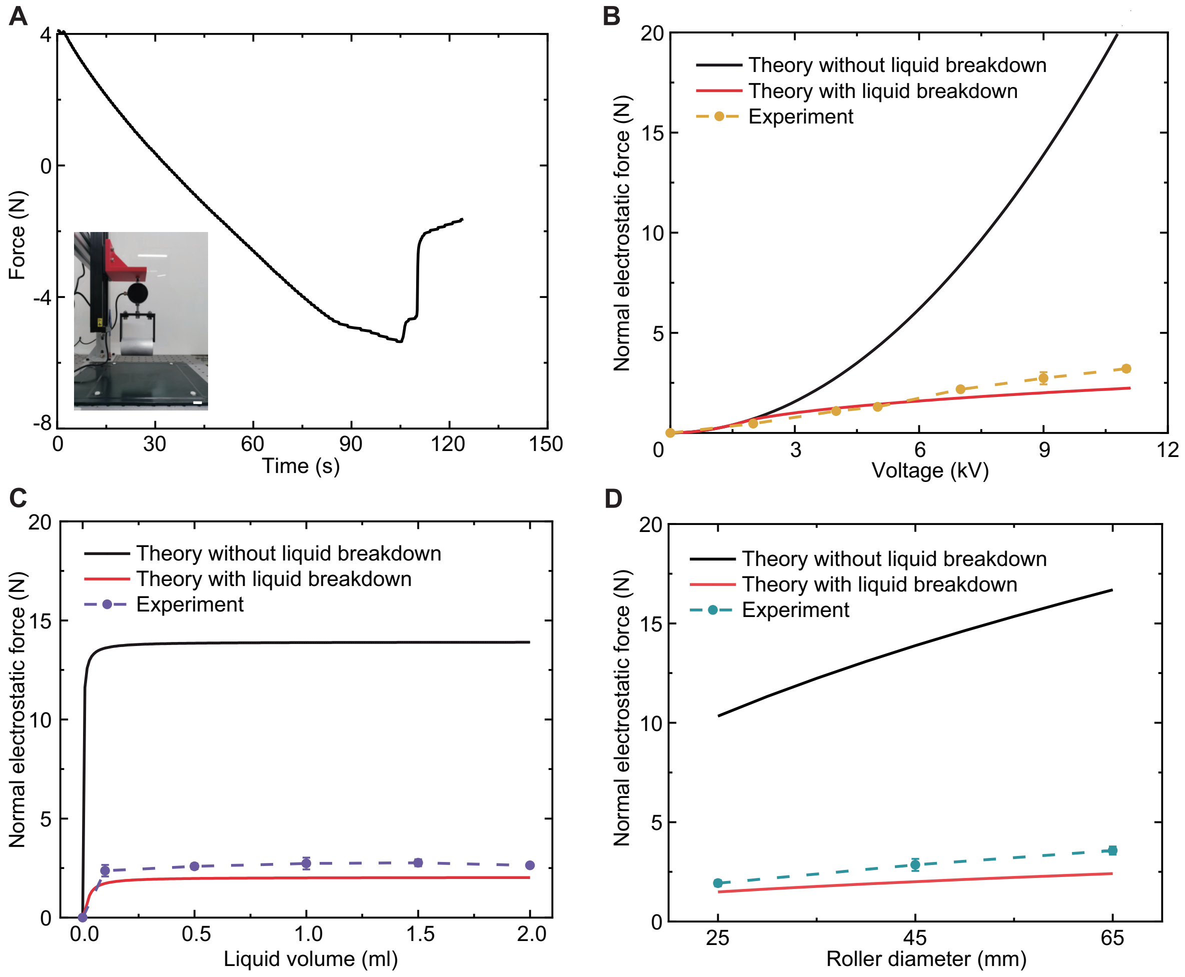

Figure S4: **Experimental and theoretical results on the normal electrostatic force of centimeter-scale rigid LAER rollers. A** Normal electrostatic force versus time obtained from experiments. The inset is the related experimental setup. **B** Effect of voltage on normal electrostatic force. **C** Effect of liquid volume on normal electrostatic force. **D** Effect of roller diameter on normal electrostatic force. Scale bar denotes 20 mm.

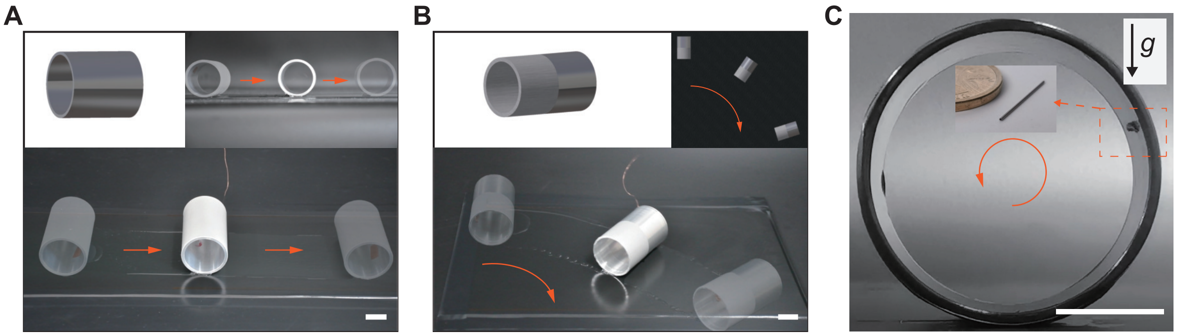

Figure S5: **Versatile moving of rigid LAER rollers. A** Forward moving of large-scale rigid LAER rollers. **B** Turing of large-scale rigid LAER rollers with non-uniform surface roughness. **C** Moving on curved surface of submillimeter-scale rigid LAER rollers. Scale bars denote 20 mm.

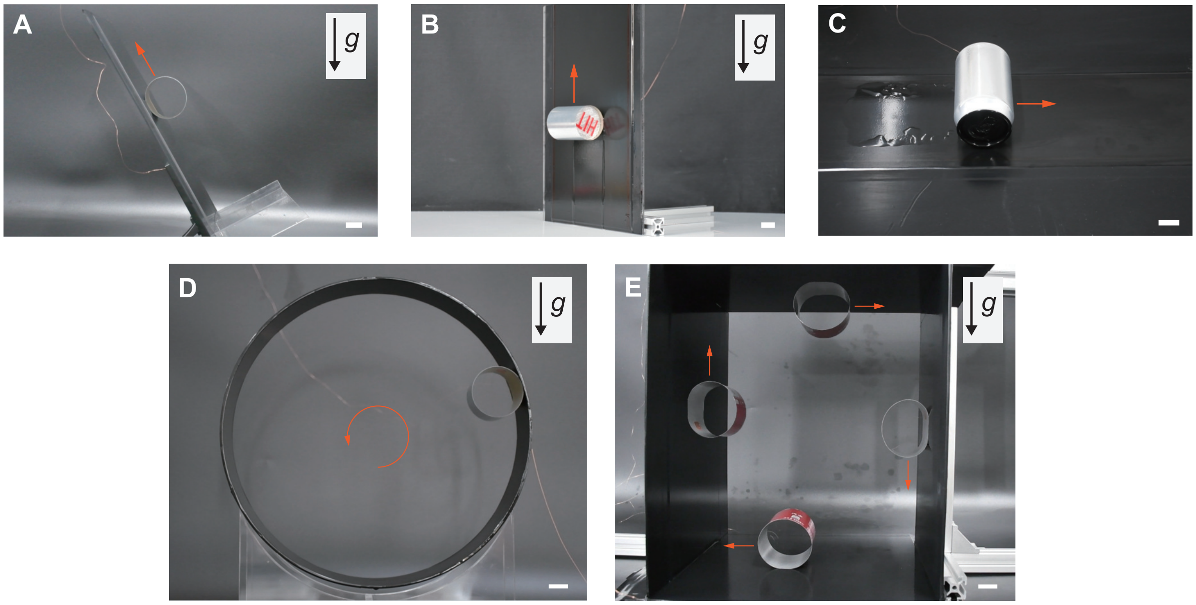

Figure S6: **Versatile moving of flexible LAER rollers. A** Moving on 60° slopes of centimeter-scale flexible LAER rollers. **B** Moving on vertical walls of centimeter-scale flexible LAER rollers. **C** Forward moving with a load-to-weight ratio of ∼121 of an unopened can. **D** Moving on curved surfaces of centimeter-scale flexible LAER rollers. **E** Plane-to-plan transitioning of centimeter-scale flexible LAER rollers. Scale bars denote 20 mm.

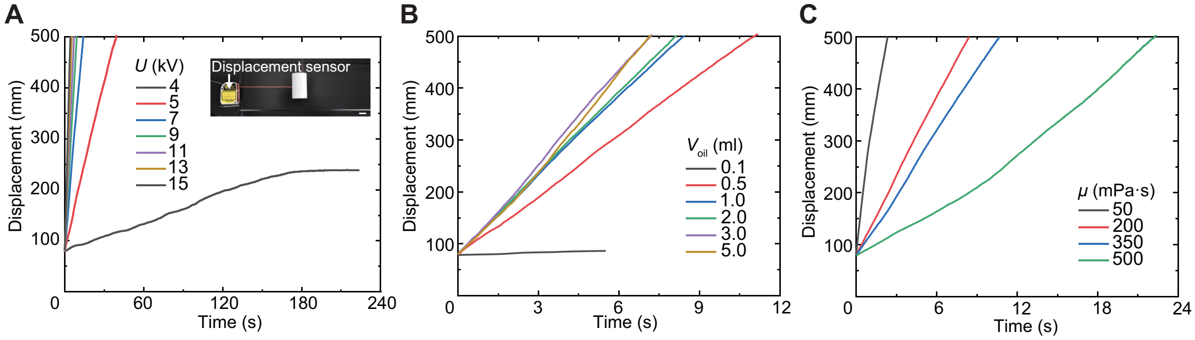

Figure S7: **Displacement-time curves of large-scale rigid LAER rollers. A** Displacement-time curves for different applied voltages. **B** Displacement-time curves for different liquid volumes. **C** Displacement-time curves for different liquid viscosities. Scale bar denotes 20 mm.

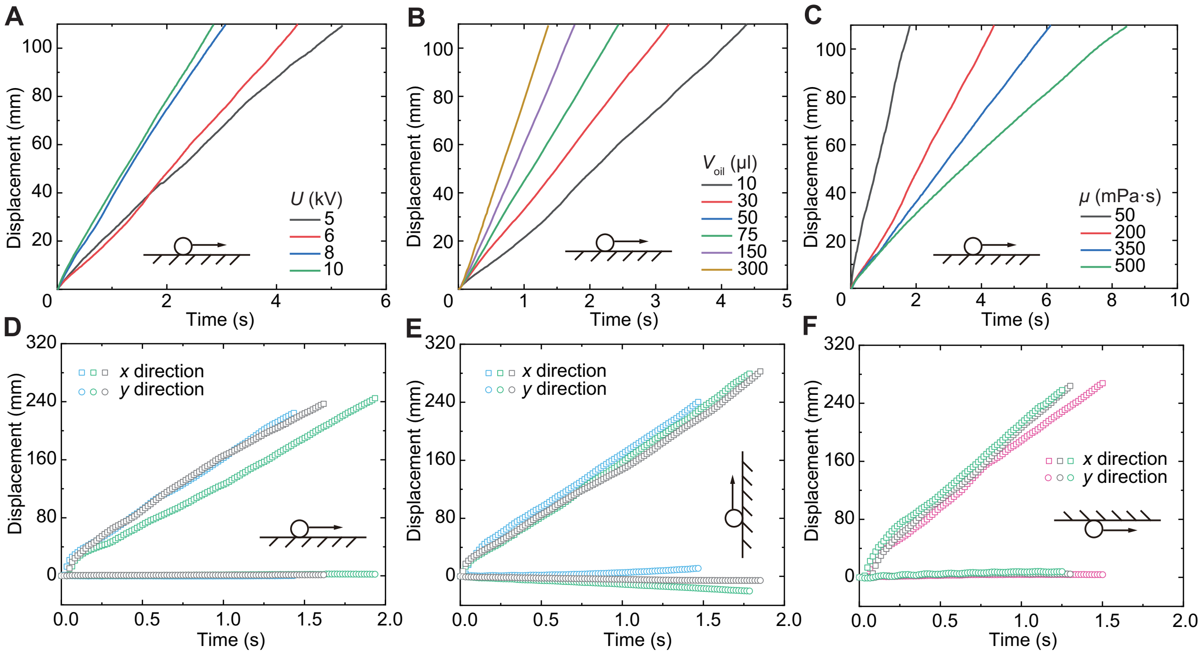

Figure S8: **Displacement-time curves of submillimeter-scale rigid LAER rollers. A** Displacement-time curves for different applied voltages. **B** Displacement-time curves for different liquid volumes. **C** Displacement-time curves for different liquid viscosities. **D** Three repeated displacement-time curves corresponding to the fastest crawling speed. **E** Three repeated displacement-time curves corresponding to the fastest vertical climbing speed. **F** Three repeated displacement-time curves corresponding to the fastest inverted climbing speed. In D-F, different colors represent different repeated experiments.

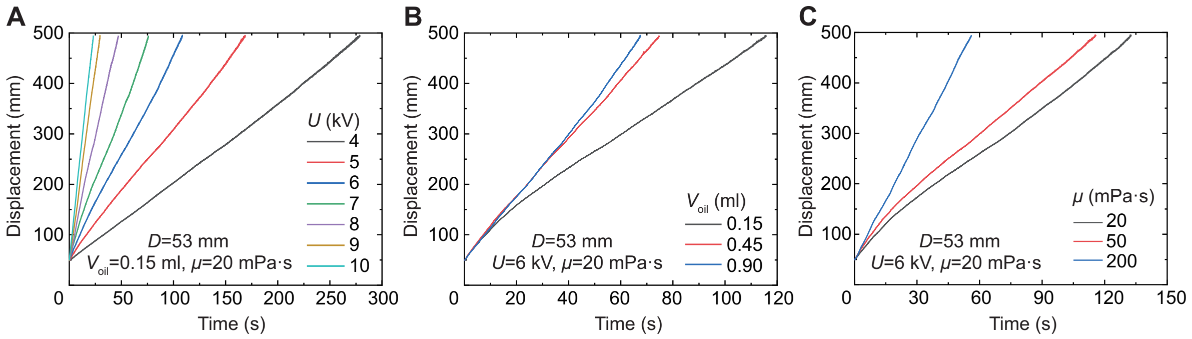

Figure S9: **Displacement-time curves of large-scale flexible LAER rollers. A** Displacement-time curves for different applied voltages. **B** Displacement-time curves for different liquid volumes. **C** Displacement-time curves for different liquid viscosities.

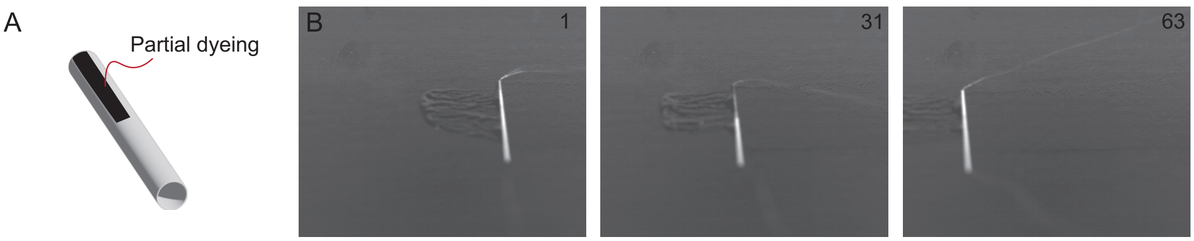

Figure S10: **Slow-motion (0.05×) playback of the rolling process of the submillimeter-scale LAER roller. A** Partially dyed sub-millimeter LAER rollers. **B** Snapshots of the rolling process captured by a high-speed camera. From left to right are frames 1, 31, and 63. The capture frame rate is 500 fps.

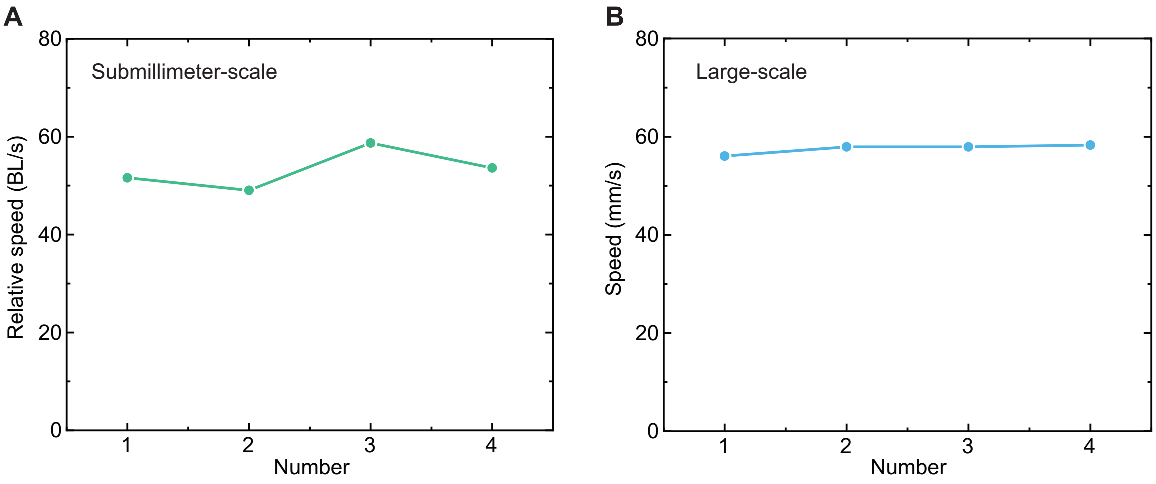

Figure S11: **The effect of the residual liquid of roller surface on the forward moving speed of rigid LAER rollers. A** The effect of the residual liquid of roller surface on the speed of submillimeter-scale LAER rollers. **B** The effect of the residual liquid of roller surface on the speed of large-scale LAER rollers.

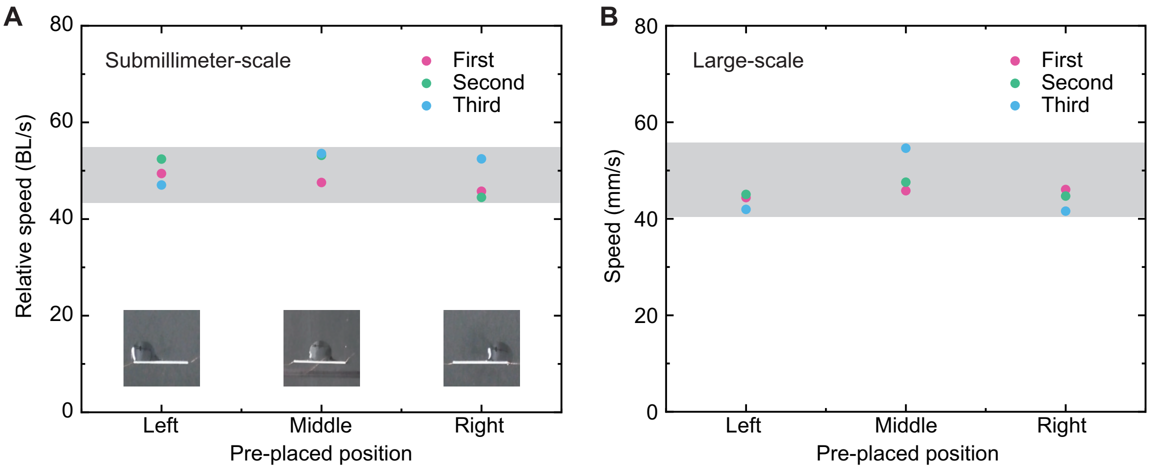

Figure S12: **The effect of the liquid pre-placed position on the forward moving speed of LAER rollers. A** The effect of the liquid pre-placed position on the speed of submillimeter-scale LAER rollers. **B** The effect of the liquid pre-placed position on the speed of large-scale LAER rollers.

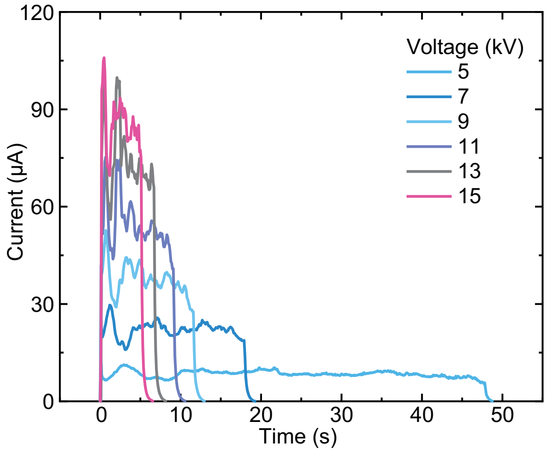

Figure S13: **Current as a function of time of large-scale rigid LAER roller for the same travel distance 500 mm.**

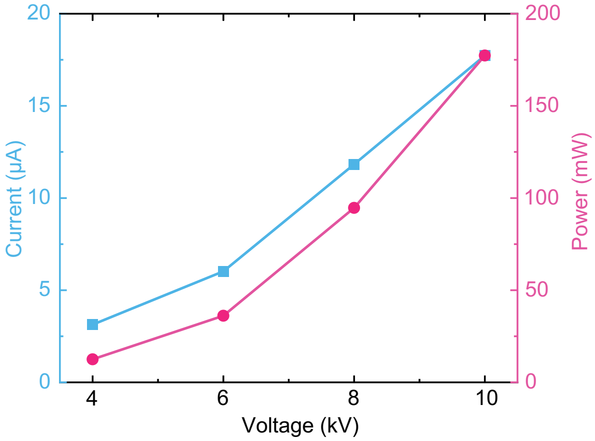

Figure S14: **Average current and power consumption as a function of applied voltage for large-scale flexible LAER rollers.**

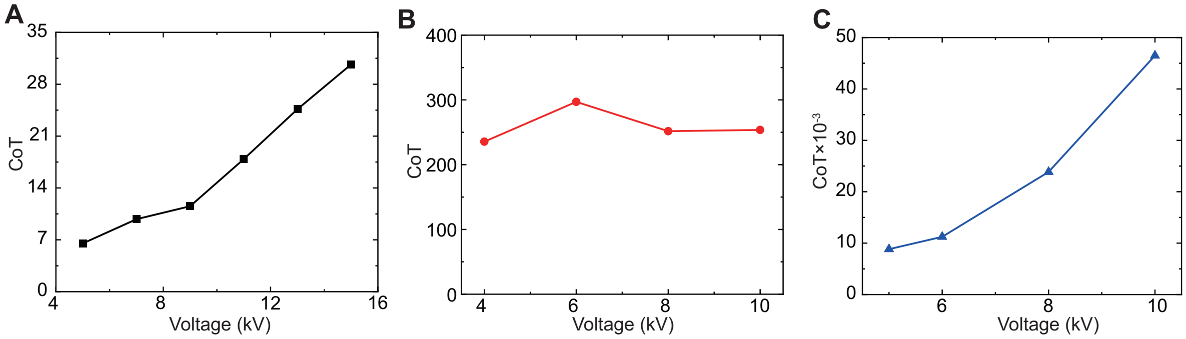

Figure S15: **COT as a function of voltage for LAER rollers. A** COT as a function of voltage for large-scale rigid LAER rollers. **B** COT as a function of voltage for large-scale flexible LAER rollers. **C** COT as a function of voltage for submillimeter-scale rigid LAER rollers.

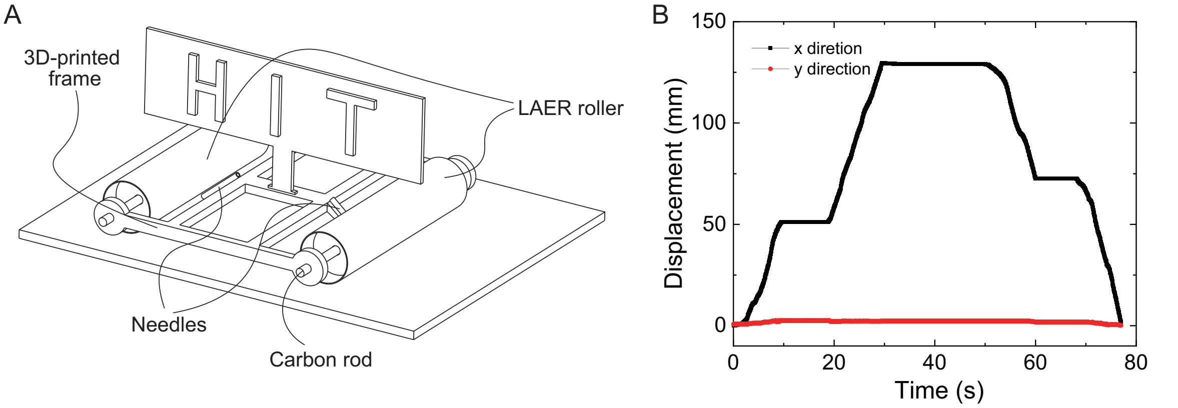

Figure S16: **Design scheme and displacement-time curve for the serial LAER robot. A** Isometric view of the serial LAER robot. **B** Displacement-time curve of the serial LAER robot.

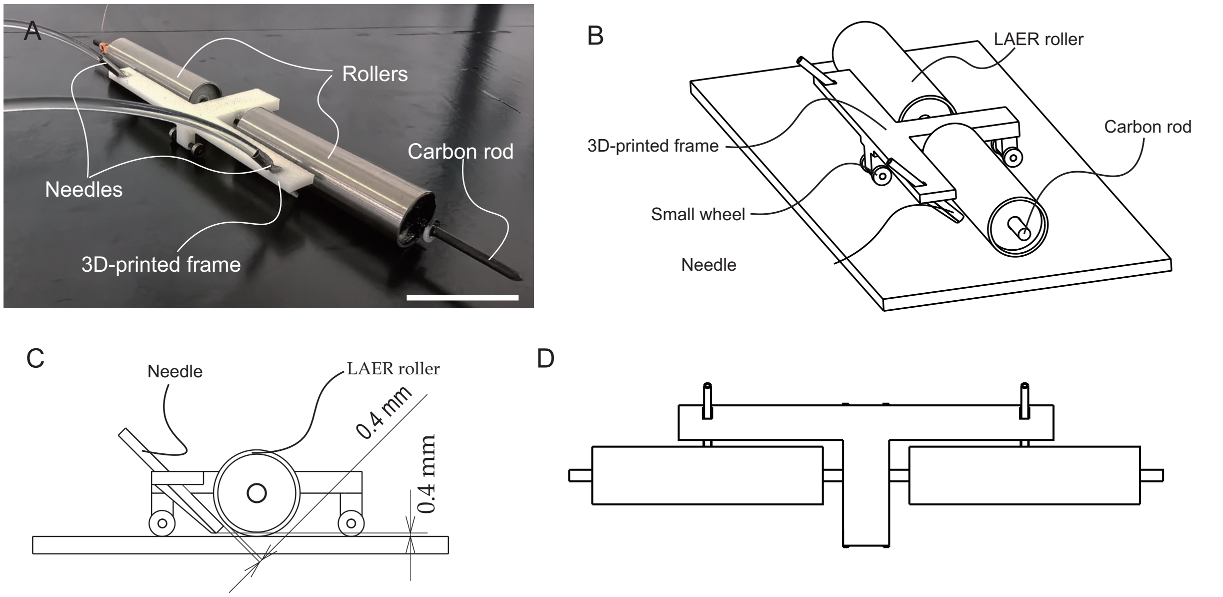

Figure S17: **Design scheme of the parallel LAER robot. A** Prototype of the parallel LAER robot. **B** Isometric view of parallel LAER robot. **C** Cross-sectional view of the parallel LAER robot. **D** Top view of the parallel LAER robot. Scale bar denotes 20 mm.

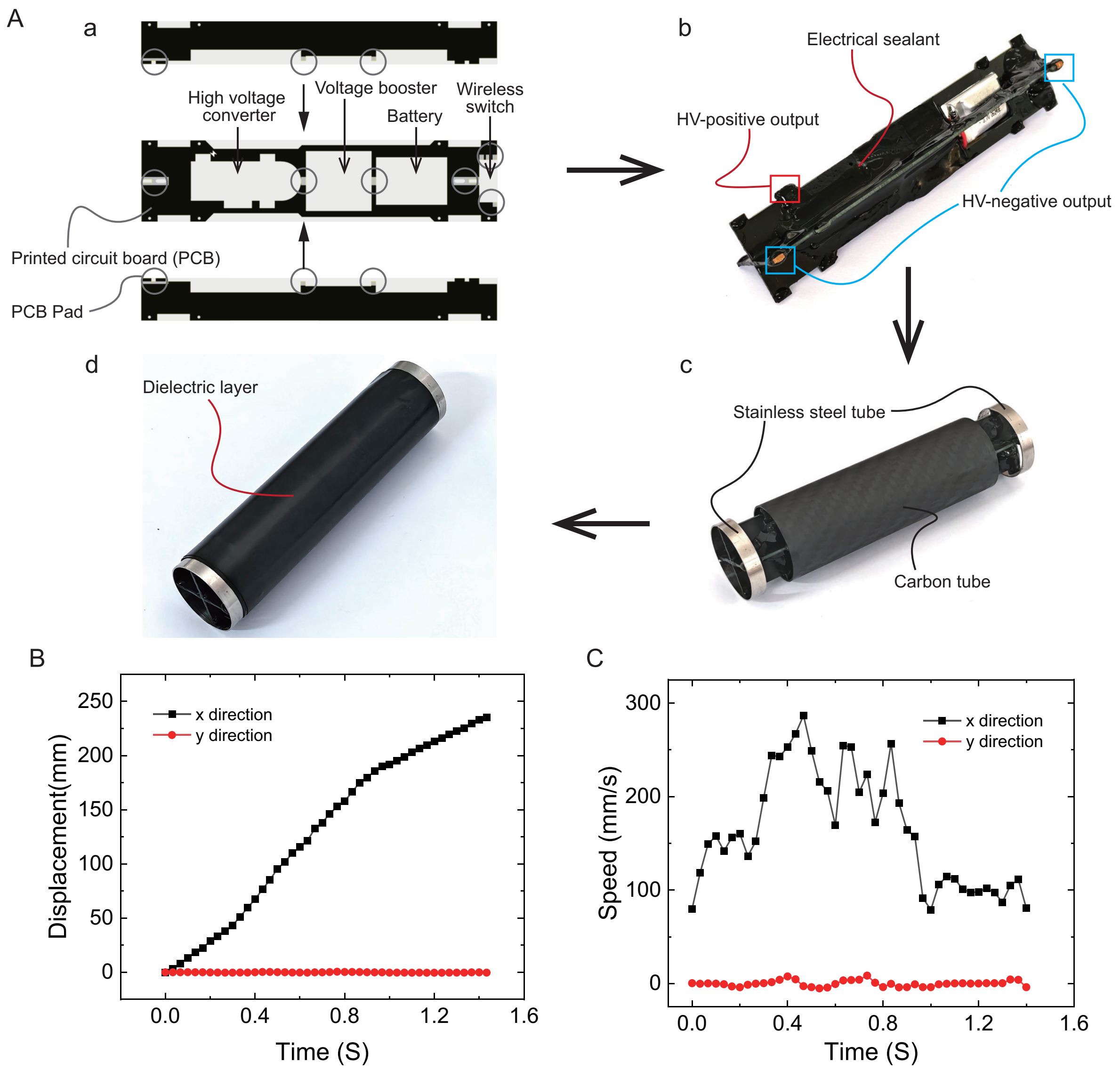

Figure S18: **Preparation process and locomotive performance of the single-wheeled untethered LAER robot. A** Preparation process of the single-wheeled untethered LAER robot. **B** Displacement-time curve of the single-wheeled untethered LAER robot. **C** Speed-time curve of of the single-wheeled untethered LAER robot.

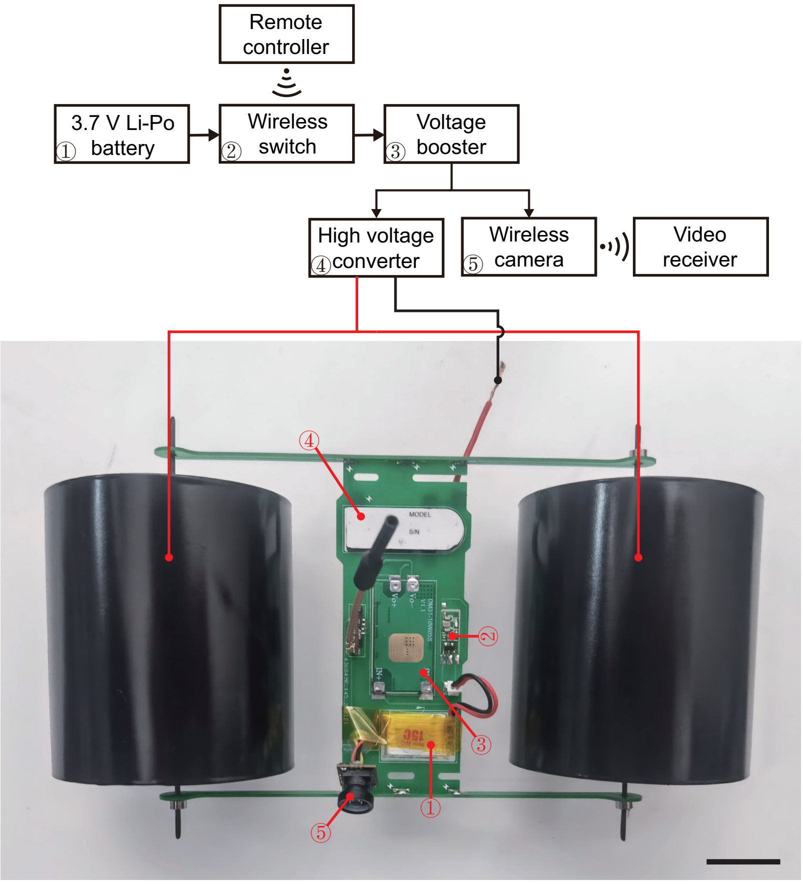

Figure S19: **Electronic design of the two-wheeled untethered LAER robot for environmental monitoring and detection.** Scale bar denotes 20 mm.

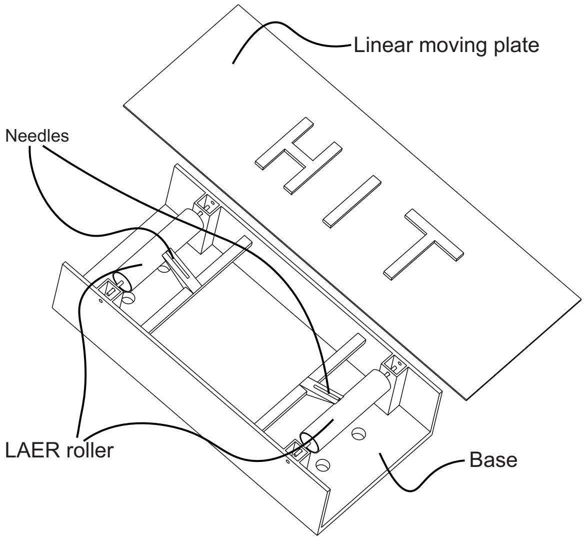

Figure S20: **Design scheme for the LAER-based linear actuator.**

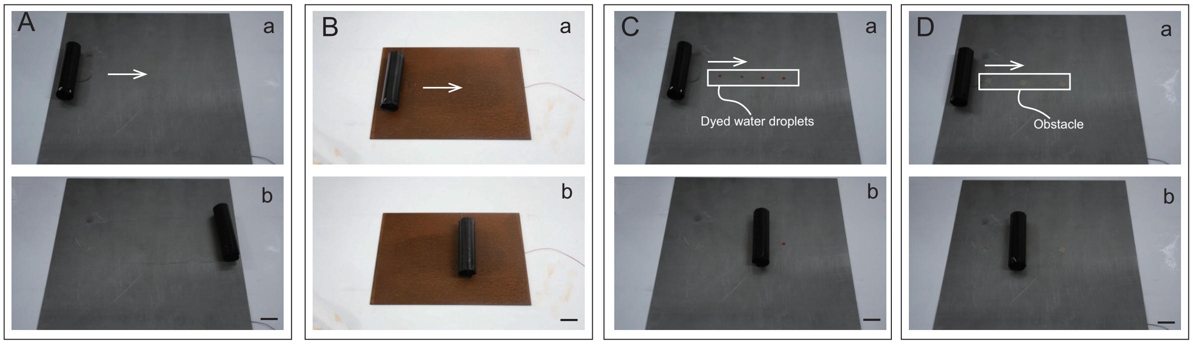

Figure S21: **Qualitative observations on environmental robustness of LAER rollers. A** Stainless steel plate as a reference case. **B** Rusty iron plate. **C** Stainless steel plate with water droplets. **D** Stainless steel plate with obstacles.

Table S1: **Components of the untethered LAER robot.**

Description	Model	Weight (g)
Li-Po battery	3.7 V/100 mAh	3.036
Voltage booster module	DM31-10W05S	5.176
High voltage converter	AG60P-5	6.254
Wireless switch	DC3V-4.2V1A, ANNTEM	0.220
Image transmission module	E7082VMV12, ZENCHANSI	1.600
Camera	800TVL	1.200
Wheel 1	\	10.934
Wheel 2	\	10.869
Frame	\	8.292
Total weight	\	47.581

Caption for Movie S1. Forward moving of LAER rollers.

The forward moving of a centimeter-scale rigid roller with D = 45.0 mm and t_{ring} = 2 mm under V_{oil} = 0.90 ml and μ = 200 mPa·s and U = 7.50 kV.

The forward moving of a centimeter-scale flexible roller with D = 53.0 mm and $t_{ring} \approx$ 0.1 mm under V_{oil} = 0.45 ml and μ = 20 mPa·s and U = 6.00 kV.

The forward moving of a submillimeter-scale rigid roller with D = 0.5 mm and t_{ring} = 0.1 mm under V_{oil} = 10 µl and μ = 350 mPa·s and U = 6.00 kV.

Caption for Movie S2. Turning and circular moving of centimeter-scale rigid LAER rollers.

The turning of a centimeter-scale rigid roller with D = 45.0 mm and t_{ring} = 3 mm with different surface textures, relative rough and smooth surface texture, under V_{oil} = 2.00 ml, μ = 200 mPa·s and U = 9.00 kV. The direction of the turning was the rougher side.

The circular moving a centimeter-scale rigid roller with a tapered structure, one end is D = 45.0 mm and the other end is D = 25.0 mm and t_{ring} = 3 mm under V_{oil} = 1.50 ml, μ = 200 mPa·s and U = 8.00 kV.

Caption for Movie S3. Circular climbing of LAER rollers.

The circular climbing of a large-scale rigid roller with D = 45.0 mm and t_{ring} = 1 mm under V_{oil} = 2.00 ml, μ = 350 mPa·s and U = 8.50 kV.

The circular climbing of a large-scale flexible roller with D = 53.0 mm under U = 6.00 kV, μ = 20 mPa·s and V_{oil} = 1.50 ml.

The circular climbing of a small-scale rigid roller with D = 0.5 mm and t_{ring} = 0.1 mm under U = 5.50 kV, μ = 350 mPa·s and V_{oil} = 10 µl.

Caption for Movie S4. Slope, vertical, inverted climbing and plane-to-plane transitioning of LAER rollers.

A large-scale flexible roller with D = 53.0 mm climbed the slopes of 30° and 60° under U = 5.50 kV, V_{oil} = 0.80 ml and μ = 20 mPa·s.

A large-scale flexible roller was obtained by wire electrical discharge machining of Coca Cola cans, and had D = 66.2 mm and W = 60 mm. It demonstrated the vertical and inverted climbing and plane-to-plane transitioning under U = 4.60 kV and μ = 20 mPa·s by sequentially controlling the four wall electrodes.

Caption for Movie S5. Rapid vertical and inverted climbing of a small-scale rigid LAER roller.

The observed maximum vertical climbing speed (~152 BL/s) of the small-scale rigid roller was demonstrated under U = 10.00 kV, μ = 50 mPa·s and V_{oil} = 10 μl.

The observed maximum inverted climbing speed (~210 BL/s) of the small-scale rigid roller was demonstrated under U = 10.00 kV, μ = 50 mPa·s and V_{oil} = 10 μl.

Caption for Movie S6. Rapid crawling of a small-scale rigid LAER roller.

The rapid moving of the small-scale rigid roller was demonstrated under U = 8.00 kV, μ = 10 mPa·s and V_{oil} = 10 μl.

The corresponding slow-motion (0.05×) playback of the above rolling process.

Caption for Movie S7. Crawling and climbing with loading of large-scale flexible LAER rollers.

An unopened Coca-Cola can (330 ml), that is readily available, moved forward by LAER at a speed of 0.12 BL/s under D = 4.75 kV, μ = 20 mPa·s and V_{oil} = 0.60 ml.

A large-scale flexible roller with D = 66.2 mm, W = 167.8 mm and t_{ring} ≈ 0.11 mm, carrying a weight of 1278.60 g (load-to-weight ratio of ~121), moved forward at a speed of 0.07 BL/s under U = 7.00 kV, μ = 50 mPa·s and V_{oil} = 1.30 ml.

A flexible roller with D = 53.0 mm, W = 75 mm and t_{ring} ≈ 0.11 mm carrying a payload of 59.63 g (load-to-weight ratio of 16.47), climbed the vertical wall at a speed of 0.60 BL/s under U = 10.00 kV, μ = 20 mPa·s and V_{oil} = 1.50 ml.

Caption for Movie S8. Reciprocating moving of serial LAER robot with liquid regulating modules.

The applied voltage was 5.00 kV, and the viscosity of the dielectric liquid was 350 mPa·s.

Caption for Movie S9. Controlled turning of parallel LAER robot with liquid regulating modules.

The applied voltage was 7.00 kV, and the viscosity of the dielectric liquid was 350 mPa·s.

Caption for Movie S10. Rapid moving of single-wheeled untethered LAER robot.

The viscosity and volume of the dielectric liquid were 10 mPa·s and 1.00 ml, respectively.

Caption for Movie S11. Reciprocating motion of the LAER-based linear actuator.

The applied voltage was 6.00 kV, and the viscosity of the dielectric liquid was 350 mPa·s.

Caption for Movie S12. Crawling of the untethered LAER robot on different conducting materials.

The crawling of the untethered LAER robot was demonstrated under μ = 50 mPa·s.

Caption for Movie S13. Vertical climbing of the untethered LAER robot on different conducting materials.

The climbing of the untethered LAER robot was demonstrated under μ = 200 mPa·s.

Caption for Movie S14. Inverted climbing of the untethered LAER robot on different conducting materials.

The inverted climbing of the untethered LAER robot was demonstrated under μ = 200 mPa·s.

Caption for Movie S15. Environmental robustness of LAER rollers.

The viscosity and volume of the dielectric liquid were 350 mPa·s and 1.00 ml, respectively. The applied voltage was 6.50 kV.
